# Extended JAZ degron sequence for plant hormone binding in jasmonate co-receptor of tomato *Sl*COI1-*Sl*JAZ

**DOI:** 10.1038/s41598-021-93067-1

**Published:** 2021-06-30

**Authors:** Rina Saito, Kengo Hayashi, Haruna Nomoto, Misuzu Nakayama, Yousuke Takaoka, Hiroaki Saito, Souhei Yamagami, Toshiya Muto, Minoru Ueda

**Affiliations:** 1 Department of Molecular and Chemical Life Sciences, Graduate School of Life Sciences, Tohoku University, Sendai, 980-8578 Japan; 2 Department of Chemistry, Graduate School of Science, Tohoku University, Sendai, 980-8578 Japan; 3 Center for Basic Education, Faculty of Pharmaceutical Sciences, Hokuriku University, Kanazawa, 920-1181 Japan

**Keywords:** Plant hormones, Jasmonic acid

## Abstract

(+)-7-*iso*-Jasmonoyl-l-isoleucine (JA-Ile) is a lipid-derived phytohormone implicated in plant development, reproduction, and defense in response to pathogens and herbivorous insects. All these effects are instigated by the perception of JA-Ile by the COI1-JAZ co-receptor in the plant body, which in *Arabidopsis thaliana* is profoundly influenced by the short JAZ degron sequence (V/L)P(Q/I)AR(R/K) of the JAZ protein. Here, we report that *Sl*JAZ-*Sl*COI1, the COI1-JAZ co-receptor found in the tomato plant, relies on the extended JAZ degron sequence (V/L)P(Q/I)AR(R/K)XSLX instead of the canonical JAZ degron. This finding illuminates our understanding of the mechanism of ligand perception by JA-Ile in this plant, and will inform both efforts to improve it by genetic modification of the *Sl*COI1-*Sl*JAZ co-receptor, and the development of the synthetic agonists/antagonists.

## Introduction

Lipid-derived (+)-7-*iso*-jasmonoyl-l-isoleucine (JA-Ile) is a major class of phytohormones implicated in plant development, reproduction, and the defense response of plants against pathogens and herbivorous insects^1,2^. Exposure of plants to external stress causes jasmonate signaling, which is triggered by the production of JA-Ile from jasmonic acid (JA)^3,4^. Perception of JA-Ile by the COI1-JAZ co-receptor causes upregulation of JA-responsive genes, leading to the expression of JAZ (JASMONATE ZIM-DOMAIN) repressor protein –a hub of jasmonate signaling^5,6^.

The JAZ protein contains two major functional domains: Jas and TIFY. In the absence of JA-Ile, the Jas domain causes JAZ to interact with various transcription factors (TFs), including the master regulator MYC2^7^. The TIFY domain of JAZ interacts with domain C of the adaptor protein Novel Interactor of JAZ (NINJA), the ethylene-responsive element binding factor-associated amphiphilic repression (EAR) domain of which recruits co-repressor TOPLESS (TPL)^8^. These interactions constitute a transcriptional repression machinery consisting of the MYC2-JAZ-NINJA-TPL complex, which represses the expression of JA-responsive gene. In the presence of JA-Ile, JAZ also plays an important role in releasing the repression of TFs. The Jas motif is also important for protein–protein interactions with COI1, a subunit of SCF^COI1^E3 ubiquitin ligase, which forms the COI1-JA-Ile-JAZ co-receptor complex with the JA-Ile ligand^9^. JA-Ile causes COI1-JAZ co-receptor formation and subsequent ubiquitination and degradation of JAZ to derepress the expression of JA-responsive genes^10–12^.

Recently, it has become clear that the *JAZ* gene functions redundantly; however, each *JAZ* subfamily gene is also responsible for its own unique function^13–17^, and differences in the sequence of the Jas motif profoundly influence the unique function of each JAZ. This is partly because the function of JAZ depends on how strongly and with which of the many TFs it interacts^13^. The Jas motif also affects the lifespan of each JAZ in plant cells (through the affinity between JAZ and COI1), which in turn determines the duration of effect of each JAZ’s unique function^13^.

**Figure 1:**
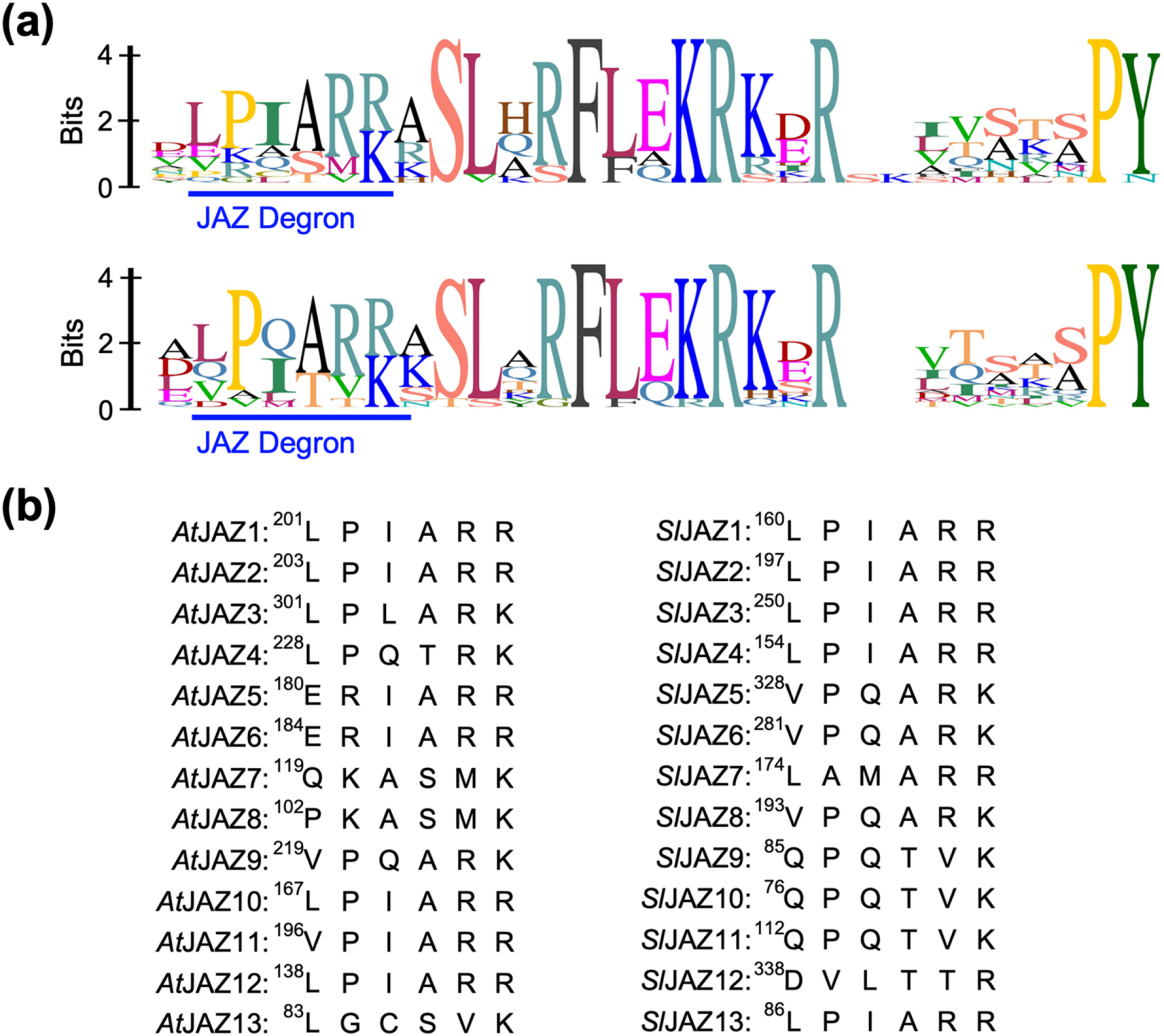
(**a**) Sequence logos of the Jas motif of *At*JAZs (top) or *Sl*JAZs (bottom). Sequence logos were created by Geneious 2019.2.1 software (TOMY DIGITAL BIOLOGY CO.,LTD., Japan). (**b**) Canonical JAZ degron sequences of *At*JAZs (left) and *Sl*JAZs (right).

The crystal structure of the COI1-JA-Ile-JAZ1 degron peptide, which also includes inositol pentakisphosphate (InsP5), revealed that the short JAZ degron sequence in the Jas motif is responsible for COI1-JA-Ile-JAZ1 complex formation, based on the discovery of a hydrogen bond network between the JAZ1 degron sequence LPIARR and COI1-JA-Ile complex^18^. JAZ belongs to the TIFY protein family, but only the JAZ subfamily incorporates the Jas motif, including the short canonical degron sequence LPIAR(R/K) necessary for binding with JA-Ile (Fig. 1a,b). The JAZ degron sequence is highly conserved among plant species, except for the two non-canonical degron sequences IPMQRK of *Fa*JAZ1 found in the strawberry plant (*Fragaria* × *ananassa*), and MPIARK of *Ec*JAZ1 found in finger millet *Eleusine coracana*^19–21^.

Slight differences in the *Arabidopsis* JAZ degron sequences (identity 37.0% in Fig. 1a) are thought to account for the difference in affinity (*K*_D_) between JAZ and COI1-JA-Ile^22^. However, the 12 JAZs of *Solanum lycopersicum* have a remarkably well-conserved JAZ degron sequence (V/L)P(Q/I)AR(R/K) (identity 46.6% in Fig. 1a), suggesting their affinities (*K*_D_) for the *Sl*COI1-JA-Ile complex are very similar (Fig. 1a). Here, we report the first comprehensive study of the affinity of the *Sl*JAZ-*Sl*COI1 co-receptor for JA-Ile, and demonstrate that the perception of JA-Ile depends on the extended JAZ degron sequence (V/L)P(Q/I)AR(R/K)XSLX. This use of an extended degron sequence (instead of the canonical short JAZ degron sequence) accounts for the different affinities of *Sl*JAZ and *Sl*COI1-JA-Ile.

## Results

### The affinities of *Sl*JAZ1-11/13 for *Sl*COI1-JA-Ile are different

All the *TIFY* sequences of the JAZ proteins of *Solanum lycopersicum* have been previously reported: 19 *TIFY* genes including 12 canonical *JAZ* (*Sl*JAZ1-11/13) and non-canonical *SlJAZ12* genes are encoded in the *Solanum lycopersicum* genome^23–25^. The Jas motifs of the 12 canonical *SlJAZs* are remarkably similar to the *Arabidopsis* consensus Jas motif SLX_2_FX_2_KRX_2_RX_5_PY, and the JAZ degron sequence (V/L)P(Q/I)AR(R/K), wherein the hydrophobic L/V is conjugated with P(Q/I)AR and followed by basic R/K is conserved in eight out of 12 *SlJAZs* (*Sl*JAZ1-6/8/13) (Figs. 1a,b). Accordingly, *Sl*COI1-*Sl*JAZ1-6/8/13 are expected to perceive JA-Ile with equal affinity, whereas *Sl*COI1-*Sl*JAZ9-11 (which incorporate a non-canonical JAZ degron) are not expected to perceive it at all. Accordingly, we examined the affinities of 12 *Sl*JAZ proteins for the *Sl*COI1-JA-Ile complex by pull-down assay.

**Figure 2:**
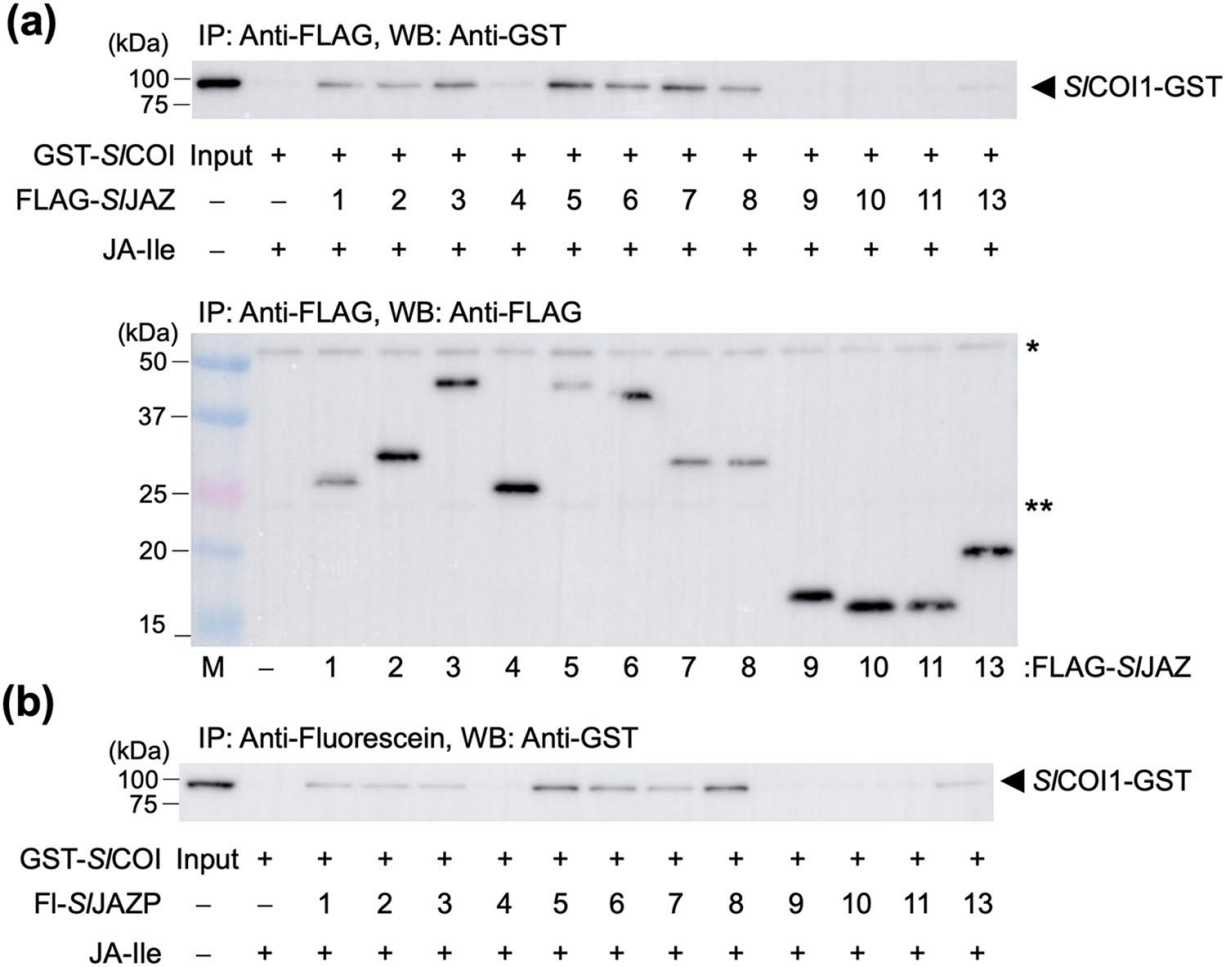
(**a**) Pull down assay of GST-*Sl*COI1 with FLAG-*Sl*JAZ (full-length proteins) in the presence of JA-Ile (100 nM). GST-*Sl*COI1 bound to FLAG-*Sl*JAZ proteins was pulled down with anti-FLAG antibody and Protein G magnetic beads, and analyzed by immunoblotting (top: anti-GST-HRP conjugate for detection of GST-*Sl*COI1, bottom: anti-FLAG antibody and anti mouse-IgG HRP conjugate for detection of FLAG-*Sl*JAZs). *or **show the bands derived from heavy chain or light chain of the anti-FLAG antibody. (**b**) Pull down assay of GST-*Sl*COI1 with Fl-*Sl*JAZPs in the presence of JA-Ile (100 nM). GST-*Sl*COI1 bound to Fl-*Sl*JAZPs was pulled down with anti-fluorescein antibody and Protein G magnetic beads, and analyzed by immunoblotting (anti-GST-HRP conjugate for detection of GST-*Sl*COI1).

*SlJAZ* genes were cloned from the tomato cultivar Micro-Tom and their FLAG-tag-fused proteins FLAG-*Sl*JAZ1-11/13 expressed using the wheat germ-derived cell-free protein expression system (Supplementary Fig. S1). GST-fused *Sl*COI1 (GST-*Sl*COI1) was also expressed in Sf9 cultured insect cells (Supplementary Fig. S2). *Arabidopsis* ASK protein was co-expressed to improve the stability of *Sl*COI1^26^. As expected, *Sl*JAZ1-3/5–8 but not FLAG-*Sl*JAZ9-11 pulled-down GST-*Sl*COI1 in the presence of 100 nM JA-Ile (Fig. 2a and Supplementary Fig. S3). However, *Sl*JAZ4/13 (which incorporates the same canonical JAZ degron LPIARR as *Sl*JAZ1-3) could not pull-down the GST-*Sl*COI1 under the same conditions (Figs. 1b and 2a). Identical results were obtained using coronatine (COR), a naturally occurring phytotoxin and known structural mimic of JA-Ile, suggesting that COR is perceived by the co-receptor in place of JA-Ile in a similar manner to JA-Ile (Supplementary Fig. S4). These results indicate that sequences other than the highly conserved *Sl*JAZ degron in full-length JAZ affect the perception of JA-Ile by the *Sl*COI1-*Sl*JAZ co-receptor.

**Figure 3:**
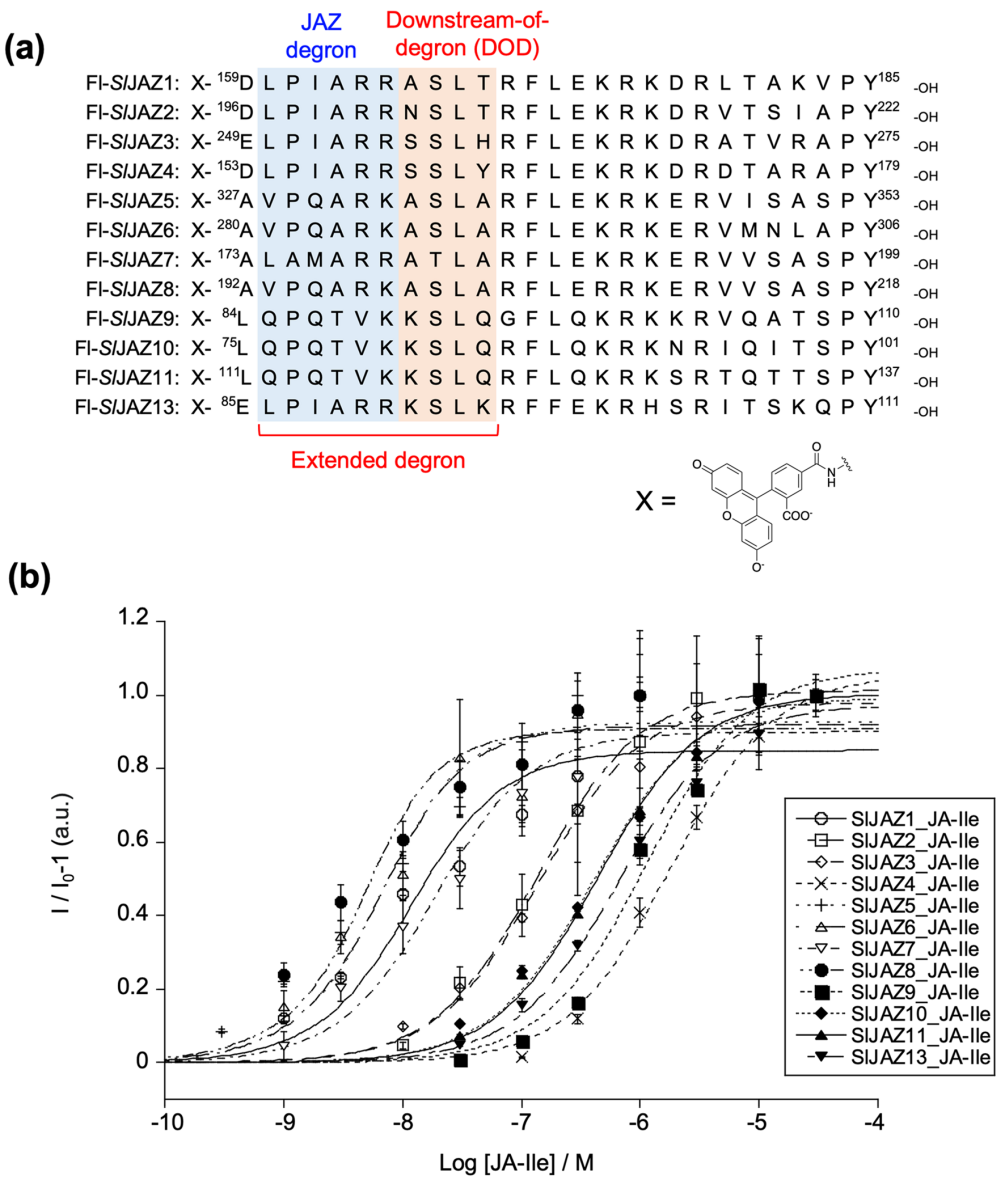
(**a**) Chemical structures of fluorescein-tagged *Sl*JAZ1-11/13 degron short peptides (*Sl*JAZP1-11/13). Canonical JAZ degron sequences are shown in blue and down-stream-of-degron (DOD) sequence are in orange. (**b**) AlphaScreen assays using Fl-*Sl*JAZPs and GST-*Sl*COI1 with JA-Ile (0–30 µM). Experiments were performed in triplicate to obtain mean and S.D. (shown as error bars). The figure was created by KaleidaGraph 4.1.1 (Synergy, Software, US).

To quantitatively examine the effect of the exo-degron sequence, we designed and synthesized fluorescein-tagged *Sl*JAZ1-11/13 degron short peptides (*Sl*JAZP1-11/13), each 27 amino acids in length (Fig. 3a and Supplementary Figs. S5–S6), based on previous work on *Arabidopsis* COI1-JAZ^22^. The pull-down assay using the GST-*Sl*COI1 and Fl-*Sl*JAZ degron peptides in the presence of increasing concentrations of JA-Ile yielded very similar results to those obtained using full-length *Sl*JAZs (Fig. 2b and Supplementary Fig. S7). Therefore, the affinity of full-length JAZ was confirmed to depend on the sequence in these short peptides. The observed affinities were quantitatively assessed in AlphaScreen luminescence proximity assays using *Sl*JAZPs and GST-*Sl*COI1 in the presence of 0–30 µM JA-Ile (Fig. 3b and Supplementary Fig. S8)^7,27^, and found to be in good accordance with the results obtained by pull-down assay: 20 > *K*_d_ for *Sl*JAZ1/5-8 of strong affinity, 150 > *K*_d_ for *Sl*JAZ2/3 of weak affinity, *K*_d_ > 400 for *Sl*JAZ4/9-11/13 of no/little affinity (Table 1). Similar results were obtained using COR (Supplementary Figs. S9 and S10).

**Table 1:** *K*_d_ values calculated from AlphaScreen analyses.

*Sl*COl1-*Sl*JAZ	K_d_/nM	
	JA-lle	COR
*Sl*JAZ1	9.2	4.9
*Sl*JAZ2	134	12.1
*Sl*JAZ3	136	19.3
*Sl*JAZ4	1776	130
*Sl*JAZ5	4.2	0.64
*Sl*JAZ6	4.1	0.76
*Sl*JAZ7	16.2	0.23
*Sl*JAZ8	2.2	0.39
*Sl*JAZ9	1107	44.7
*Sl*JAZ10	402	89.7
*Sl*JAZ11	430	62.4
*Sl*JAZ13	637	80.7

### Hormone perception relies on extended JAZ degron sequences in tomato *Sl*JAZs

Here, we focused on the relationship between the degron sequences of *Sl*JAZPs and their *K*_d_ values. The short JAZ degron sequence (L/V)P(Q/I)AR(R/K) was highly conserved in *Sl*JAZP1-6/8/13 (Fig. 3a). *Sl*JAZP5/6/8, which incorporate the JAZ degron sequence VPQARK, all have strong affinity for *Sl*COI1-JA-Ile. In contrast, remarkable differences in affinity were observed for *Sl*JAZP1-4/13, whose degron sequence is LPIARR: only *Sl*JAZP1 showed a moderate affinity for *Sl*COI1-JA-Ile; the others had weak/no affinity. Among *Sl*JAZ1-4/13, the difference can be found in the downstream-of-degron (DOD) sequence XSLX (Fig. 3a). This strongly suggests that sequences more extended than the canonical JAZ degron influence the affinity of *Sl*JAZs for *Sl*COI1-JA-Ile.

**Figure 4:**
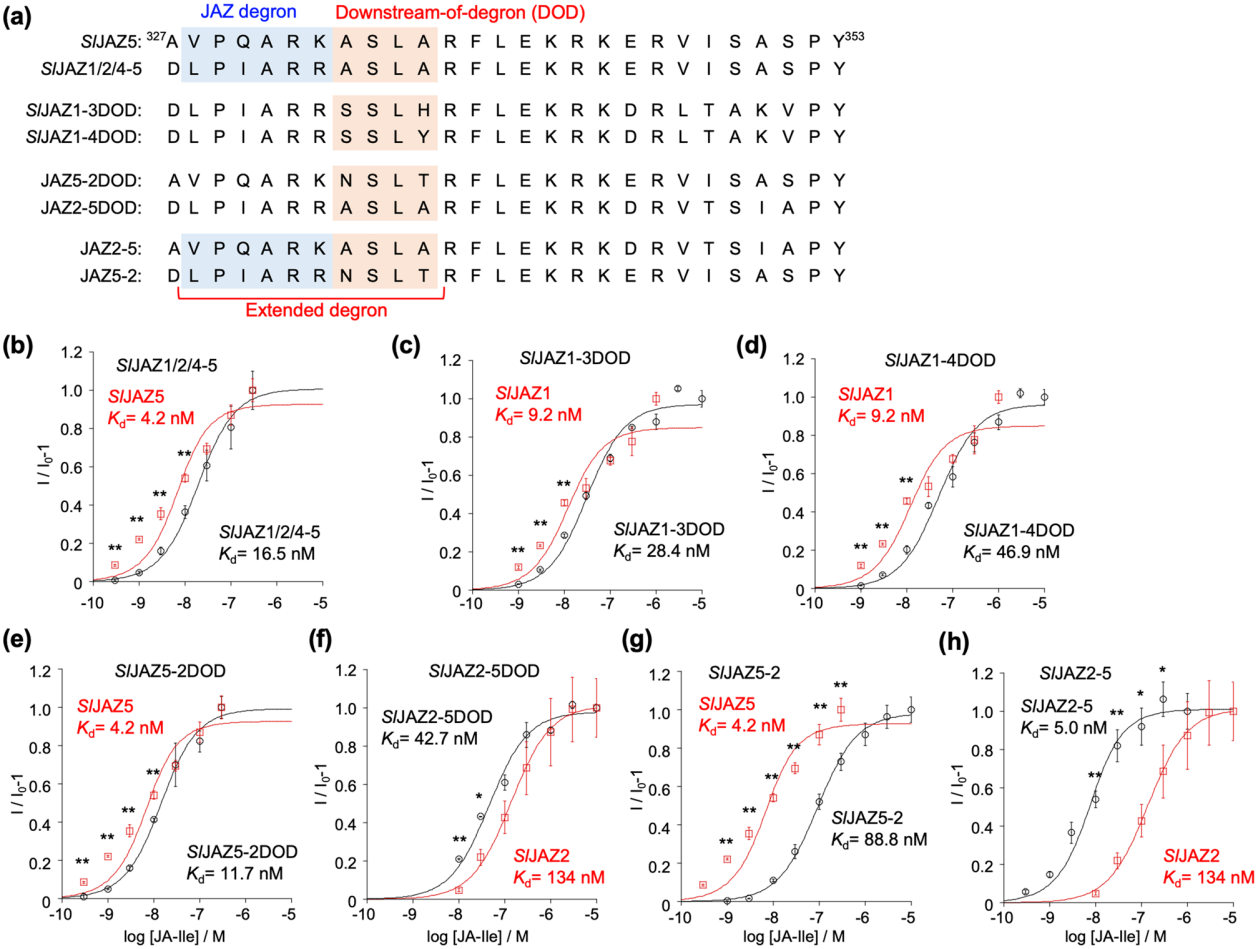
(**a**) Design of the swapped *Sl*JAZ peptides (*Sl*JAZ5, 1/2/4-5, 1-3DOD, 1-4DOD 5-2DOD, 2-5DOD, 2-5, 5-2). Canonical JAZ degron sequences are shown in blue and down-stream-of-degron (DOD) sequence are in orange. (**b**) AlphaScreen assay using GST-*Sl*COI1 and Fl-*Sl*JAZP1/2/4-5 (black circle) or Fl-*Sl*JAZ5 (red square) with JA-Ile (0–300 nM). (**c**–**h**) Alphascreen assays of swapped Fl-*Sl*JAZPs (**c**: SlJAZ1-3DOD, **d**: 1-4DOD, **e**: 5-2DOD, **f**: 2-5DOD, **g**: 5–2, **h**: 2–5) to study the extended degron sequence. Signal intensity changes of AlphaScreen of swapped Fl-*Sl*JAZPs and GST-*Sl*COI1 upon addition of JA-Ile (0–10 µM). Black circles show the results of swapped *Sl*JAZPs and red squares those of the corresponding natural *Sl*JAZPs, respectively. Experiments were performed in triplicate to obtain mean and S.D. (shown as error bars). Significant differences were evaluated by Students’ T-test (*p < 0.05, **p < 0.01). These figures were created by KaleidaGraph 4.1.1 (Synergy, Software, US).

To confirm the effect of the exo-JAZ-degron sequence within *Sl*JAZPs on their affinity, we prepared chimeric *Sl*JAZPs incorporating swapped sequences and submitted them to the AlphaScreen assay. First, we swapped the two JAZ-degron sequences VPQARK of *Sl*JAZP5/6/8 and LPIARR of *Sl*JAZP1-4/13 to examine the effect of the JAZ degron sequence on affinity. The N-terminal region of the high-affinity peptide *Sl*JAZP5 including JAZ-degron VPQARK was swapped with that of the moderate/low-affinity *Sl*JAZP1/4 including JAZ-degron LPIARR, to give the swapped peptide *Sl*JAZP1/2/4-5 (Fig. 4a, Supplementary Figs. S11 and S12). Then, we examined whether the difference in the JAZ degron sequence of *Sl*JAZs affected the affinity with *Sl*COI1-JA-Ile (Fig. 4b and Supplementary Fig. S13a). As shown in Fig. 4b, the high affinity of *Sl*JAZP5 was moderately decreased by swapping with *Sl*JAZP1/4 (*K*_d_ = 4.2 nM for *Sl*JAZP5 to *K*_d_ = 16.5 nM for *Sl*JAZP1/2/4-5). The effect of swapping was moderate, and the complete swapping of their affinities did not occur. This result suggested that the differences in JAZ degron alone cannot fully account for the difference in their affinities, which must therefore be influenced by exo-JAZ-degron sequences in addition to the canonical JAZ degron.

To examine the effect of the DOD sequence, we studied *Sl*JAZP1-4/13 which incorporate the same JAZ degron sequence LPIARR and alternative DOD sequence XSLX. We focused on three *Sl*JAZPs: *Sl*JAZP1, which is of strong affinity (*K*_d_ = 9.2 nM); *Sl*JAZP3, of moderate affinity (*K*_d_ = 136 nM); and *Sl*JAZP4, of no affinity (*K*_d_ = 1776 nM). We prepared *Sl*JAZP1-3DOD and *Sl*JAZP1-4DOD, wherein the DOD sequence of *Sl*JAZP1 was swapped with those of *Sl*JAZP3 and *Sl*JAZP4, respectively (Fig. 4a, and Supplementary Figs. S11–12). As shown in Fig. 4c,d (and Supplementary Fig. S13b, c), their affinities for *Sl*COI1-JA-Ile were moderately decreased (*K*_d_ = 28.4 nM for *Sl*JAZP1-3DOD and *K*_d_ = 46.9 nM for *Sl*JAZP1-4DOD). This result confirmed that the DOD sequence, in addition to the JAZ degron, affected the affinity between *Sl*JAZ and *Sl*COI1-JA-Ile. Next, we examined whether the DOD sequence also affected the affinity of *Sl*JAZP5/6/8 with another JAZ degron sequence VPQARK. As *Sl*JAZP5/6/8 has the same DOD sequence ASLA, we replaced the DOD of *Sl*JAZP5 of strong affinity (*K*_d_ = 4.2 nM) with that of *Sl*JAZ2 of weak affinity (*K*_d_ = 134 nM). We prepared DOD swapped peptides *Sl*JAZP5-2DOD (ASLA of *Sl*JAZ5 to NSLT of *Sl*JAZ2) and *Sl*JAZP2-5DOD (NSLT of *Sl*JAZ2 to ASLA of *Sl*JAZ5) (Fig. 4a, and Supplementary Figs. S11–12). Their affinities for *Sl*COI1-JA-Ile were affected by this swapping (*K*_d_ = 11.7 nM for *Sl*JAZP5-2DOD and *K*_d_ = 42.7 nM for *Sl*JAZP2-5DOD, Fig. 4e,f and Supplementary Fig. S13d, e). This result suggested that the DOD sequence in *Sl*JAZ5/6/8 also affects affinity for *Sl*COI1-JA-Ile.

We also swapped the extended JAZ degron sequence including the JAZ-degron and DOD to prepare swapped peptides of *Sl*JAZP5-2 (DLPIARRNSLT of SlJAZ2 into AVPQARKASLA of SlJAZ5) and *Sl*JAZP2-5 (AVPQARKASLA of SlJAZ5 into DLPIARRNSLT of SlJAZ2) (Fig. 4a, and Supplementary Figs. S11–12). Their affinities with *Sl*COI1-JA-Ile were completely replaced by this sequence swapping (*K*_d_ = 88.8 nM for *Sl*JAZP5-2 and *K*_d_ = 5.0 nM for *Sl*JAZP2-5, respectively, Fig. 4g,h and Supplementary Fig. S13f, g). This result confirmed that the extended JAZ degron sequence including JAZ-degron and DOD affects the affinity of in SlJAZ with *Sl*COI1-JA-Ile.

From all of these results, we concluded that extended-JAZ-degron sequences, VPQARKASLA or LPIARRXSLX, determine the affinity of *Sl*JAZs with *Sl*COI1-JA-Ile.

### In silico simulation demonstrated the role of extended JAZ degron sequences in *Sl*COI1-JA-Ile-*Sl*JAZ complex formation

**Figure 5:**
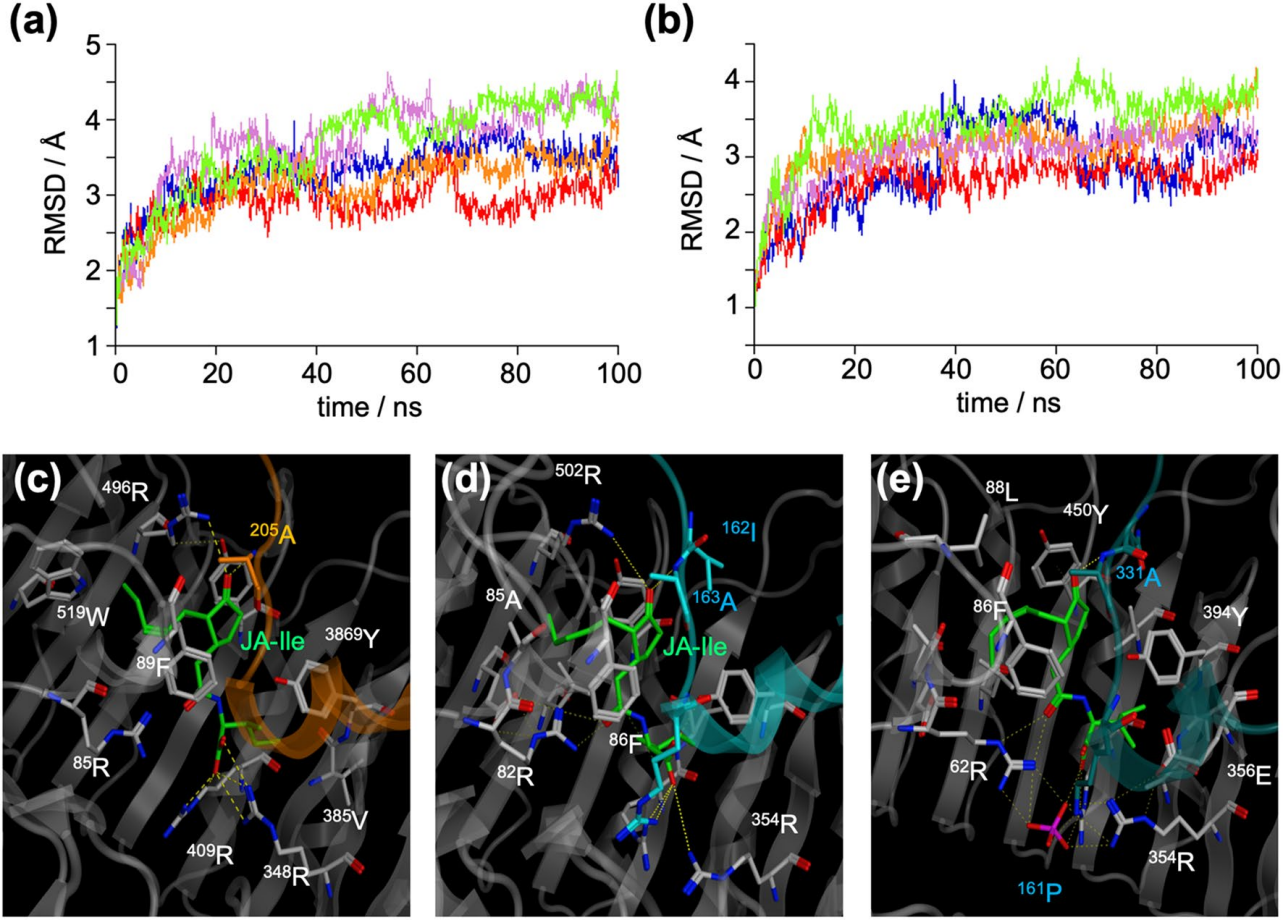
(**a**,**b**) The RMSDs of backbone atoms of the complex of *Sl*COI1-JA-Ile-*Sl*JAZ1 (**a**) and *Sl*COI1-JA-Ile-*Sl*JAZ5 (**b**) as a function of time. Each plot depicted in blue, red, orange, purple, or green was obtained from the five independent MD simulation. (**c**) The reported structure of the ligand binding pocket of *At*COI1-JA-Ile-*At*JAZ1 (PDB ID: 3OGL). (**d**,**e**) The representative structure of the ligand binding pocket of *Sl*COI1-JA-Ile-*Sl*JAZ1 (**d**) and that of *Sl*COI1-JA-Ile-*Sl*JAZ5 (**e**), which was obtained by homology modeling and MD simulation. Hydrogen bonds are shown as yellow dotted lines. The RMSD plot was created by KaleidaGraph 4.1.1 (Synergy, Software, US), and the images of the 3D structures were created by MOE 2020.09.

**Figure 6:**
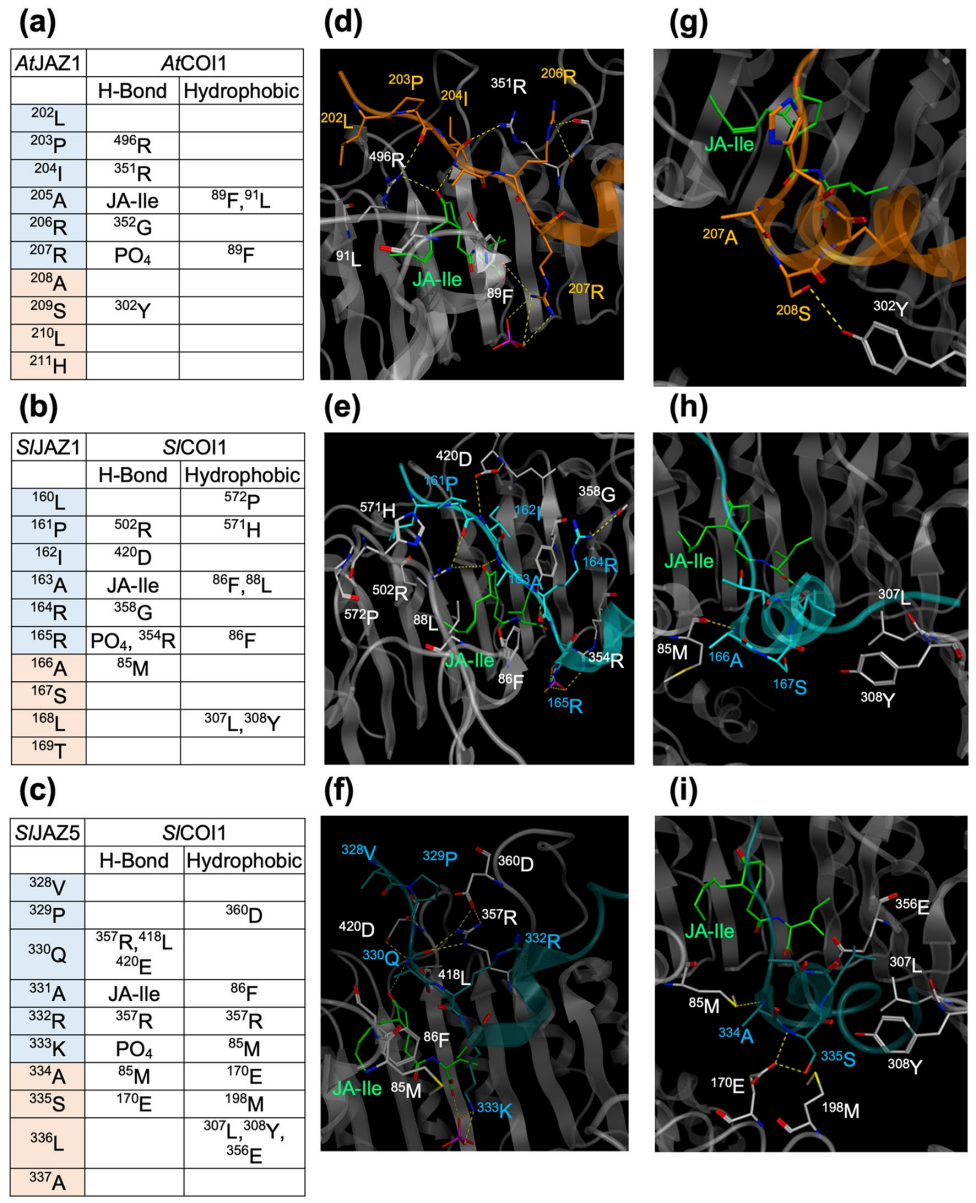
In silico analyses of *At*COI1-JA-Ile-*At*JAZ1 (PDB ID: 3OGL, **a**,**d**,**g**), *Sl*COI1-JA-Ile-*Sl*JAZ1 (**b**,**e**,**h**), and *Sl*COI1-JA-Ile-*Sl*JAZ5 (**c**,**f**,**i**) to show the binding mode of the extended degron. (**a**–**c**) The amino acid residues forming hydrogen bonds (H-bond) or hydrophobic interaction (Hydrophobic) between COI1 and JAZ observed in the crystal structure of *At*COI1-JA-Ile-*At*JAZ1 (3OGL, **a**), MD simulation of *Sl*COI1-JA-Ile-*Sl*JAZ1 (**b**) and MD simulation of *Sl*COI1-JA-Ile-*Sl*JAZ5 (**c**). (**d**) The reported structure around the degron sequence of *At*JAZ1 in the complex of *At*COI1-JA-Ile-*At*JAZ1. (**e**,**f**) The obtained MD structure around the degron sequence of *Sl*JAZ in the complex of *Sl*COI1-JA-Ile-*Sl*JAZ1 (**e**) or *Sl*COI1-JA-Ile-*Sl*JAZ5 (**f**). (**g**) The reported structure around the DOD sequence of *At*JAZ1 in the complex of *At*COI1-JA-Ile-*At*JAZ1. (**h**,**i**) The obtained MD structure around the DOD sequence of *Sl*JAZ in the complex of *Sl*COI1-JA-Ile-*Sl*JAZ1 (**h**) or *Sl*COI1-JA-Ile-*Sl*JAZ5 (**i**). Hydrogen bonds are shown as yellow dotted lines. The images of the 3D structures were created by MOE 2020.09.

In silico docking simulation studies using the crystal structure of *Arabidopsis* COI1-JA-Ile-JAZ1 have previously enabled the study of the JA-Ile binding mode of the COI1-JAZ co-receptor of other plant species, such as *Phaseolus lunatus*, *Eleusine coracana* (L.) Gaertn, *Fragaria vesca*, and *Fragaria* × *ananassa*^20,21,28,29^*.* The contribution of the extended JAZ degron sequence to *Sl*COI1-JA-Ile-*Sl*JAZ complex formation was examined by comparing the crystal structure of *Arabidopsis* COI1-JA-Ile-JAZ1 with the in silico interaction models of *Sl*COI1-JAIle-*Sl*JAZ1 and *Sl*COI1-JA-Ile-*Sl*JAZ5. A homology model of *Sl*COI1 was obtained with the software Molecular Operating Environment (MOE) using the crystal structure of *At*COI1 (PDB ID: 3OGL) as a template (the sequence identity of *Sl*COI1 and *At*COI1 is 68.1%). *At*JAZ1 in the homology model of *Sl*COI1-JA-Ile-*At*JAZ1 was replaced with *Sl*JAZ1 or *Sl*JAZ5. The obtained models of these complexes (*Sl*COI1-JA-Ile-*Sl*JAZ1 and *Sl*COI1-JA-Ile-*Sl*JAZ5) were then used for subsequent molecular dynamics (MD) simulations. Root means square deviation (RMSD) values indicated that the structures reached equilibrium after 50 ns or more (Fig. 5a,b). There were no significant differences in the canonical JAZ degron sequence (LPIARR) interactions in *At*JAZ1/*Sl*COI1 with *At*COI1-JA-Ile/*Sl*JAZ1-JA-Ile, nor in the hydrogen-bonding network formed around JA-Ile (Figs. 5c–e, 6a–f, and Supplementary Fig. S14) in any of the three complexes. In addition, no direct interaction between the DOD sequences of *Sl*JAZs and JA-Ile was observed in *Sl*COI1-JA-Ile-*Sl*JAZ1/5. These results suggest that the DOD sequence of *Sl*JAZs does not interact with JA-Ile. Therefore the DOD sequence may enhance the *Sl*COI1-*Sl*JAZs interaction. Next, we compared the interaction between the DOD sequence and *Sl*COI1 in *Sl*COI1-JA-Ile-*Sl*JAZ1/5 with the interaction between the corresponding sequence in *At*JAZ1 (ASLH) and *At*COI1. In *At*COI1-JA-Ile-*At*JAZ1, a weak interaction through one hydrogen bond was found between the DOD sequence and *At*COI1 (Fig. 6g), whereas in *Sl*COI1-JA-Ile-*Sl*JAZ1 and *Sl*COI1-JA-Ile-*Sl*JAZ5, a strong interaction through several hydrogen bonds or hydrophobic interactions was found (Fig. 6b,c,h,i, and Supplementary Fig. S15). This indicates that the DOD sequence in *Sl*JAZ significantly contributes to the formation of the *Sl*COI1-JA-Ile-*Sl*JAZ complex, consistent with the results of the previous experiments. However, the results in Table 1 in which *Sl*COI1-*Sl*JAZ1 has a lower affinity for JA-Ile/COR than *Sl*COI1-*Sl*JAZ5 cannot be explained by the in silico docking studies because of the slight differences in their affinities.

## Discussion

JAZ functions as a repressor of numerous TFs in plant cells, and the differences in function between JAZ family proteins are profoundly affected by how strongly and with which of the many TFs each JAZ interacts. Since JAZs interact with many TFs through the Jas motif, differences in this motif can account for differences in the function of JAZs. In addition, JAZ interacts with the COI1-JA-Ile complex using the degron sequence within the Jas motif, and ubiquitinated and degraded as a substrate for E3 ubiquitin ligase. A slight difference in the degron sequence of each JAZ can profoundly impact the duration of JAZ function because it affects the lifetime of each JAZ in the plant cell. Therefore, differences in the strength of the interaction between each JAZ and the COI1-JA-Ile complex affect the turn-over of each JAZ^30^.

This study is the first comprehensive investigation into the affinity of the *Sl*JAZ-*Sl*COI1 co-receptor for JA-Ile. *Sl*JAZ9-11, having a non-canonical JAZ degron sequence QPQTVK, were found to have no affinity for *Sl*COI1-JA-Ile. However, in transcript expression of *Sl*JAZs on JA-treated Micro-Tom, *Sl*JAZ9-11 were also JA-responsive: *Sl*JAZ9/10 were weakly induced in both roots and leaves, and *Sl*JAZ11 was strongly induced in roots but weakly in leaves^23^. In *Arabidopsis*, non-canonical *At*JAZ7/8/13 lacking a conserved degron sequence do not have affinity for *At*COI1-JA-Ile and play a unique role in transcriptional repression in plant cells^31,32^. *At*JAZ10.4, an alternative splice variant of JAZ10, is involved in the negative feedback regulation of JA signaling and is responsible for the delayed repression of activated JA signals^33^. A similar function is inferred for JA-responsive *Sl*JAZ9-11, despite its lack of affinity for *Sl*COI1-JA-Ile: *Sl*JAZ9-11 may act as repressors, similar to *At*JAZ7/8/13, or the *Sl*COI1-*Sl*JAZ9-11 pairs could perceive another ligand. The same QPQTVK sequence is also observed in a JAZ member of *Solanum tuberosum*^19^.

The crystal structure of the *Arabidopsis At*COI1-JA-Ile-*At*JAZ1 complex revealed important details regarding the interaction between *At*JAZ and *At*COI1-JA-Ile complex. The short canonical degron sequence LPIARR of *At*JAZ1 overlies the top of JA-Ile binding pocket of *At*COI1, covering the JA-Ile bound to *At*COI1, and interacting with both *At*COI1 and JA-Ile. Detailed analyses revealed that each amino acid in the LPIARR sequence interacts with JA-Ile by hydrogen-bond formation (LPIARR) or hydrophobic interaction (LPIARR). Thus, any change in the conserved LPIAR(R/K) sequence will affect the affinity between *At*JAZ and *At*COI1-JA-Ile. Comparison of the amino acid sequences of the JAZ degron among the *Arabidopsis* functional JAZs showed slight differences from LPIARR of *At*JAZ1, which will be responsible for the difference in affinity for *At*COI1-JA-Ile among the *At*JAZs (*K*_d_ 7–34 for JA-Ile on fluorescence anisotropy assay)^34^. Small differences in the *Arabidopsis* JAZ degron sequences are considered responsible for the difference in their affinity.

In contrast, the degron sequence differences among functional *Sl*JAZs are smaller than those of *Arabidopsis* JAZs, but each *Sl*JAZ nevertheless binds with different affinities to *Sl*COI1-JA-Ile (Figs. 2, 3 and Table 1). In particular, significant discrepancies in the affinities of *Sl*JAZ1-4/13 for *Sl*COI1-JA-Ile were observed, even though their JAZ degron sequences are identical.

We prepared the swapped *Sl*JAZPs and found that their affinity with *Sl*COI1-JA-Ile strongly depends on the extended JAZ degron sequence of VPQARKASLA or LPIARRXSLX (Fig. 4). The short degron sequence (V/L)P(Q/I)AR(R/K) has been hypothesized to play a critical role in hormone reception due to its high degree of conservation across numerous plant species. The reported exceptions are the cases of strawberry JAZ1 (*Fa*JAZ1) of the non-canonical JAZ degron sequence IPMQRK and finger millet *Ec*JAZ1 of the non-canonical JAZ degron sequence MPIARK. Our result confirmed that tomato *Sl*JAZs employ the extended JAZ degron sequence of (V/L)P(Q/I)AR(R/K)XSLX for the perception of JA-Ile. The contribution of this extended JAZ-degron sequence in *Sl*COI1-JA-Ile-*Sl*JAZs complex formation was further studied using the in silico interaction models generated from the reported crystal structure of *Arabidopsis* COI1-JA-Ile-JAZ1 (Fig. 5). The DOD sequence in *Sl*JAZ formed a strong hydrogen bond network with *Sl*COI1, stabilizing the complex (Fig. 6). In interaction models of *Sl*COI1-JA-Ile-*Sl*JAZ1/5, the canonical JAZ degron of *Sl*JAZs retained the same interaction as that of *At*JAZ in *At*COI1-JA-Ile-*At*JAZ1^18^. Then, why do *Sl*JAZ1/2/3/4/13, which have LPIARR as a common canonical JAZ degron, have different affinities for *Sl*COI1-JA-Ile? Swapping experiments have shown that this is due to differences in the DOD sequence; when the DOD sequence of *Sl*JAZ1 was replaced with that of *Sl*JAZ3/4, the affinity was markedly decreased (*Sl*JAZ1-3/4DOD in Fig. 4a,c,d), indicating that the DOD sequence of *Sl*JAZ3/4 negatively affects the interaction with *Sl*COI1-JA-Ile. Specifically, two point-mutations in the DOD sequence of *Sl*JAZ1, A166S and T168H/Y, are presumed to negatively affect the interaction between the LPIARR sequence and *Sl*COI1-JA-Ile. This is the first report to demonstrate that sequences longer than the canonical JAZ degron are employed for hormone perception of COI1-JAZ co-receptor.

*Sl*JAZ7 is unique among *Sl*JAZs having three amino acid residues in the extended JAZ degron (^175^A, ^176^M, and ^181^T) which are not found in other *Sl*JAZs (Fig. 3a). *Sl*JAZ7 of a moderate affinity (*K*_d_ = 16.2 nM) (Table 1) has the extended JAZ degron sequence of LAMARRATLA in which the P(Q/I) in the canonical degron (V/L)P(Q/I)AR(R/K) is replaced by ^175^A^176^M and the highly conserved S in the DOD sequence XSLX is replaced by ^181^T. In the *Arabidopsis* COI1-JA-Ile-JAZ1 crystal structure, the PI sequence in the canonical JAZ degron of *At*JAZ1 plays an important role in the interaction with *At*COI1^18^. This suggests that *Sl*JAZ7 may interact with *Sl*COI1-JA-Ile in a manner different from other *Sl*JAZs. Remarkably, the extended JAZ degron sequence LAMARRATLA in *Sl*JAZ7 (belonging to the strong affinity group) is also present in the other two *Solanaceae* species*, Solanum tuberosum* and *Petunia axilaris*^19^. Moreover, the LAMARRATLA sequence is only present in dicots, particularly in *Solanaceae*^19^, suggesting that the LAMARR degron is a new functional degron in addition to the most common JAZ degrons LPIARR and VPQARK.

Here, we report that the affinity of *Sl*JAZ for *Sl*COI-JA-Ile depends on the extended degron sequence. Recently, a similar result was reported in the auxin co-receptor TIR1-AUX/IAA, in which additional, non-hormone interacting sequences of AUX/IAA re-enforcing auxin perception^35^. The role and flexibility of intrinsically disordered domains in hormone perception attract attentions of plant biologists.

## Conclusion

The affinity of *Sl*JAZ for *Sl*COI-JA-Ile depends on the extended degron sequence and not on the canonical degron sequence, based on the result of a comprehensive study of ligand perception of *Sl*JAZ-*Sl*COI1. *Sl*JAZ9-11 have no affinity for *Sl*COI-JA-Ile but are also JA-inducible^23^, which suggests that they have unique functions in plant cells, such as suppression of JA signaling. This finding illuminates our understanding of the mechanism of hormone perception in the edible tomato plant. Genetic modification of the *Sl*COI1-*Sl*JAZ co-receptor may improve hormone perception, and lead to the development of the synthetic agonists/antagonists.

## Materials and methods

All chemical reagents and solvents were obtained from commercial suppliers (Wako Pure Chemical Industries Co. Ltd., Nacalai Tesque Co., Ltd., Watanabe Chemical Industries Co. Ltd., Thermo Fisher Scientific K.K., GE Healthcare) and used without further purification. Coronatine (COR) and (+)-*7*-iso*-*JA-L-Ile (JA-Ile) were prepared as previously described^1,36,37^. DNA purification was accomplished using a GENE PERP STAR PI-80X (KURABO, Osaka, Japan). Ultraviolet (UV)-visible spectra were recorded on a UV-2600 spectrophotometer (Shimadzu, Kyoto, Japan). The AlphaScreen assay was carried out on an EnVision multimode plate reader (PerkinElmer, Inc., CA, US). SDS-PAGE and Western blotting were performed using Mini-Protean III (Bio-Rad Laboratories, Inc., US), Tras-Blot Turbo (Bio-Rad Laboratories, Inc., US), and iBind Flex (Thermo Fisher Scientific K.K., CA, US) electrophoresis apparatus. Chemiluminescent images were detected using the Amersham Imager 680 (GE Healthcare, CA, US). Reverse-phase high-performance liquid chromatography (HPLC) was performed on a PU-4180 plus with UV-4075 and MD-4010 detectors (JASCO, Tokyo, Japan). Absorbance at 220 nm and 488 nm was monitored by an MD-4010 photodiode array detector (PDA). MALDI-TOF MS analysis was performed on an Autoflex Max (Bruker Daltonics Inc., MA, US). 3D structures were constructed using MOE 2020.09 software (Chemical Computing Groups, Montreal, Canada).

### Preparation of the *Sl*COI1 and *Sl*JAZs proteins

Standard methods for cloning were used, and PCR-amplified DNA fragments were sequenced after cloning into the vectors. The plasmids of GST-fused AtCOI1 or AtASK1 (pFB-GTE-COI1 and pFB-HTB-ASK1) were obtained from Addgene (https://www.addgene.org/), and the plasmid for wheat-derived cell-free protein expression system (pEU-FLAG-GW-STOP) was a kind gift from Drs. Koji Miyamoto (Teikyo University), Kazunori Okada (The University of Tokyo), and Tatsuya Sawasaki (Ehime University). The full-length CDS of *Sl*COI1 was obtained from Osaka Prefecture University (kindly supported by Prof. Koh Aoki) and cloned into pFB-GTE-COI1, to prepare the plasmid of GST-fused *Sl*COI1. These *Sl*COI1 and *At*ASK1 proteins were co-expressed in insect cells and purified by Glutathione Sepharose 4B (GE Healthcare) according to the previous reports^18,34,38^. The full-length CDS of *Sl*JAZ2/3/5/6/7 were obtained from Osaka Prefecture University (kindly supported by Prof. Koh Aoki), and PCR-amplified and cloned into the pDONR221 vector (Invitrogen, CA, US) using BP reaction (Gateway). The coding sequences of *Sl*JAZ1/4 were isolated from *Solanum* cDNA using the primers. PCR-amplified *Sl*JAZ1/4 DNA was cloned into pENTR/D-TOPO (Thermo Fisher Scientific, USA). The coding sequences of *SlJAZ*8/9/10/11/13 were synthesized by the manufacturers (Eurofins Genomics K.K., Japan), and were cloned into the pDONR221 vector using the BP reaction. The CDS was then inserted into pEU-FLAG-GW-STOP vector using the LR reaction (Gateway) to prepare the plasmid for FLAG tag-fused *Sl*JAZ (pEU-FLAG-GW-*Sl*JAZs). These *Sl*JAZs proteins were expressed in wheat germ-derived cell-free protein expression system according to previous reports^39^, and used without purification. Cell-free translation reaction was performed according to the instruction protocol (Cell Free Sciences, Co., Ltd., Ehime, Japan) with minor modification. Briefly, the transcription reactions with pEU-FLAG-GW-*Sl*JAZs (each 1 µg) were performed at 37 °C for 5 h. The obtained mixture of mRNA was added to a solution of creatine kinase and WEPRO7240, to prepare the translation mixture, carefully transferred to the bottom of a well containing translation buffer to form the bilayer, and then incubated at 15 °C for 20 h in a 96-well plate. The obtained protein mixture was centrifuged (20,000*g* for 15 min at 4 °C) and the supernatant was used for the pulldown experiments without any purification.

### Synthesis of Fl-*Sl*JAZPs

All *Sl*JAZ peptides were prepared using the fully automated microwave peptide synthesizer Initiator + Alstra (Biotage Ltd, North Carolina, US) starting from Fmoc-Tyr-Wang resin (90 µm) as previously reported, with minor modifications^34^. A representative protocol is as follows. The resin was swollen in DMF at 70 °C for 20 min. The Fmoc protecting group was removed by treating with 20% piperidine in DMF twice. Amino acid coupling was accomplished by mixing the resin with Fmoc protected amino acids (3 eq), *O*-(1*H*-Benzotriazol-1-yl)-*N*,*N*,*N′*,*N'*-tetramethyluronium hexafluorophosphate (HBTU, 3 eq), 1-Hydroxy-*1H*-benzotriazole hydrate (HOBt·H_2_O) (3 eq), and DIPEA (6 eq) in DMF, and subjecting it to microwave irradiation at 50 °C for either 30 min (Fmoc-Arg-OH) or 10 min (all others). After the peptide had been fully elongated, the resin was mixed with 5-carboxy-fluorescein diacetate (3 eq), HBTU (5 eq) and DIPEA (5 eq) in DMF and incubated at r.t. for 2 h. After the reaction, the peptide was deprotected by stirring in TFA solution at r.t. for 1.5 h (deprotection of SlJAZ6 and 7 was performed using TFA solution containing thioanisole, anisole, and 1,2-ethanedithiol, for the avoidance of methionine oxidation). The reaction mixture was purified by HPLC using a Develosil ODS-HG-5 column (Φ 4.6 × 250 mm) eluting with a linear gradient (CH_3_CN (0.05% TFA):H_2_O (0.05% TFA) = 20:80 (5 min) to 50:50 (35 min)) to afford fluorescein-conjugated *Sl*JAZ peptide. After lyophilization, conjugated *Sl*JAZ peptide was dissolved in sterilized water to prepare the stock solution. The concentrations of the stock solution were determined by their absorbance at 494 nm in 0.1 N NaOH aqueous solution using a molar extinction coefficient of 75,000 M^−1^ cm^−1^. The purity of each peptide was confirmed by HPLC analyses, and these were characterized by MALDI-TOF MS as follows;

- Fl-SlJAZ1: *m/z* [M + H]^+^ calcd for 3571,92, found 3571.92
- Fl-SlJAZ2: *m/z* [M + H]^+^ calcd for 3573.86, found 3573.86
- Fl-SlJAZ3: *m/z* [M + H]^+^ calcd for 3623.92, found 3623.90
- Fl-SlJAZ4: *m/z* [M + H]^+^ calcd for 3651.85, found 3651.85
- Fl-SlJAZ5: *m/z* [M + H]^+^ calcd for 3429.81, found 3429.80
- Fl-SlJAZ6: *m/z* [M + H]^+^ calcd for 3500.83, found 3500.83
- Fl-SlJAZ7: *m/z* [M + H]^+^ calcd for 3594.97, found 3594.99
- Fl-SlJAZ8: *m/z* [M + H]^+^ calcd for 3443.80, found 3443.76
- Fl-SlJAZ9: *m/z* [M + H]^+^ calcd for 3515.88, found 3515.84
- Fl-SlJAZ10: *m/z* [M + H]^+^ calcd for 3656.97, found 3656.99
- Fl-SlJAZ11: *m/z* [M + H]^+^ calcd for 3605.90, found 3605.89
- Fl-SlJAZ13: *m/z* [M + H]^+^ calcd for 3729.97, found 3729.98
- Fl-SlJAZ1/2/4-5: *m/z* [M + H]^+^ calcd for 3500.87, found 3500.84
- Fl-SlJAZ1-3DOD: *m/z* [M + H]^+^ calcd for 3623.92, found 3623.93
- Fl-SlJAZ1-4DOD: *m/z* [M + H]^+^ calcd for 3649.93, found 3649.92
- Fl-SlJAZ2-5DOD: *m/z* [M + H]^+^ calcd for 3500.84, found 3500.84
- Fl-SlJAZ5-2DOD: *m/z* [M + H]^+^ calcd for 3502.82, found 3502.82
- Fl-SlJAZ2-5: *m/z* [M + H]^+^ calcd for 3429.81, found 3429.80
- Fl-SlJAZ5-2: *m/z* [M + H]^+^ calcd for 3573.86, found 3573.87

### Pulldown assay

All chemicals (JA-Ile or COR) were dissolved in ethanol to generate 10 mM stock solutions and diluted with 20% ethanol aq. for preparation of 100 μM stock solutions. For the pull-down experiments using full-length *Sl*JAZ proteins, purified GST-COI1 (5 nM), FLAG-tagged *Sl*JAZ (each 20 µL of the translation mixture), and the ligands (COR or JA-Ile) in 350 µL of incubation buffer (50 mM Tris–HCl buffer, pH 7.8, 100 mM NaCl, 20 mM 2-mercaptoethanol, 10% glycerol, 0.1% Tween20, 100 nM inositol-1,2,4,5,6-pentakisphosphate (IP5)) were combined with anti-FLAG antibody (0.2 µL, Sigma Aldrich, F1804, clone M2), and incubated for 10–15 h at 4 °C with rotation. After incubation, the samples were combined with SureBeads Protein G (10 µL in 50% incubation buffer slurry, Bio-Rad). After 3 h incubation at 4 °C with rotation, the samples were washed three times with 350 µL of fresh incubation buffer. The washed beads were resuspended in 35 µL of SDS-PAGE loading buffer containing dithiothreitol (DTT, 100 mM). After heating for 10 min at 60 °C, the samples were subjected to SDS-PAGE and analyzed by western blotting. The bound GST-COI1 protein was detected using anti-GST HRP conjugate (RPN1236, GE Healthcare, 5000-fold dilution in blocking buffer (Nakalai tesque, Inc., Japan). FLAG-JAZ proteins were detected using anti-FLAG antibody (1000-fold dilution in blocking buffer) and anti-mouse IgG-HRP antibody (Southern Biotech. Inc., Birmingham, US, 1031-05, 20,000-fold dilution in blocking buffer). Three independent replicates using proteins purified at different times were done with similar results (Supplementary Fig. S3).

For the pull-down experiments using fluorescein-tagged *Sl*JAZ peptides (Fl-*Sl*JAZps), purified GST-COI1 (5 nM), Fl-*Sl*JAZp (10 nM), and JA-Ile (1 µM) in 350 µL of incubation buffer were combined with anti-fluorescein antibody (0.2 µL, GeneTex, CA, US), and incubated for 10–15 h at 4 °C with rotation. After incubation, the samples were combined with SureBeads Protein G (10 µL in 50% incubation buffer slurry, Bio-Rad). The washing and eluting protocols were same as the pulldown experiments using full-length JAZ proteins. Three independent replicates using proteins and peptides purified at different times were done with similar results (Supplementary Fig. S7).

### AlphaScreen assay

AlphaScreen experiments were performed at 25 °C in the incubation buffer. 15 μL of the reaction mixture containing the incubation buffer, 5 nM *Sl*COI1, 10 nM Fl-*Sl*JAZPs and various concentrations of COR or JA-Ile was added to a 1/2 Area AlphaPlate-96 (PerkinElmer), and then incubated for 1 h at 4 °C. Then, 10 μL of a detection mixture containing incubation buffer, 0.1 μL of FITC-coated donor beads, 0.1 μL of GST-coated acceptor beads was added to each well. Finally, the mixture was incubated for 12 h and the luminescence signals were detected using the Envision 2105 Multimode Plate Reader (PerkinElmer). The experiment was repeated three times, and the data are presented as average values with standard deviation. To clarify the difference in the affinity of the ligands with each COI1/JAZ co-receptor, the results of the AlphaScreen assay are shown with the normalized signal intensity change (Figs. 3, 4, and Supplementary Fig. S9) along with the raw signal intensity (Supplementary Figs. S8, S10, and S13)^40^.

### In silico analyses

The homology modeling of SlCOI1 was obtained based on the crystal structure of *At*COI1–JA-Ile–*At*JAZ1 (PDB ID: 3OGL). The structure preparation program MOE 2020.09 was used to deduce the structures of the absent residues (residues 550–562) of *At*COI1. The model structures of *Sl*JAZ1, and 5 were constructed by mutating residues of the *At*JAZ1 peptide of the complex.

MD simulations of *Sl*COI1-JA-Ile-*Sl*JAZ1 and *Sl*COI1-JA-Ile-*Sl*JAZ5 were performed under conditions of constant temperature and pressure (*T* = 300 K, *P* = 1 atm). A Parrinello–Rahman type thermostat^41^ and Nosé-Hoover barostat^42^ were used to control the system temperature and pressure, respectively. The Amber14SB^43^ and generalized amber force field (gaff)^44^ were assigned for the protein/peptide and the ligand molecule, respectively. The TIP3P model^45^ was used for water solvent. The cutoff length for van der Waals (vdW) and coulomb interactions in real space was 12 Å. The particle mesh Ewald (PME) method^46^ was used for the estimation of the coulomb interactions. The time step for integration of equations of motions was 2 fs. All MD calculations were done by GROMACS2018 program^47^.

Snapshot structures of *Sl*COI1-JA-Ile-*Sl*JAZ1 and *Sl*COI1-JA-Ile-*Sl*JAZ5 were sampled every 10 ps. First, we performed 10 ns MD simulations for energy minimization/equilibration of the systems, and subsequently conducted five independent 100 ns MD simulations (total 500 ns) for each system. The system equilibrations of the protein/peptide and the ligand were monitored by the root means square displacement (RMSD) values as the simulation time step. Ligand binding modes were confirmed to be little changed in all MD simulations. The last snapshot structures were used as the representative structures to investigate the binding forms of the complex.

## Supplementary Information

Supplementary Information.
