# Supplementary material for: Extended JAZ degron sequence for plant hormone binding in jasmonate co-receptor of tomato SlCOI1-SlJAZ
Source: Sci Rep. 2021 Jun 30;11:13612. doi: 10.1038/s41598-021-93067-1 (PMC8245654; doi:10.1038/s41598-021-93067-1)
Supplement: Supplementary file 1 — Supplementary Information. [file 41598_2021_93067_MOESM1_ESM.pdf]

Supplementary Information for *Sci. Rep.*:

Extended JAZ degron sequence for plant hormone binding in jasmonate co-receptor of tomato *Sl*COI1-*Sl*JAZ

Authors:

Rina Saito[1], Kengo Hayashi[2], Haruna Nomoto[2], Misuzu Nakayama[2], Yousuke Takaoka[2], Hiroaki Saito[3], Souhei Yamagami[1], Toshiya Muto[2], and Minoru Ueda*[1,2]

Affiliations:

[1]Department of Molecular and Chemical Life Sciences, Graduate School of Life Sciences, Tohoku University, Sendai 980-8578, Japan

[2]Department of Chemistry, Graduate School of Science, Tohoku University, Sendai 980-8578, Japan

[3] Center for Basic Education, Faculty of Pharmaceutical Sciences, Hokuriku University, Kanazawa, 920-1181, Japan

*Correspondence: minoru.ueda.d2@tohoku.ac.jp

Contents

Figure S1-S19

Table S1

Experimental section

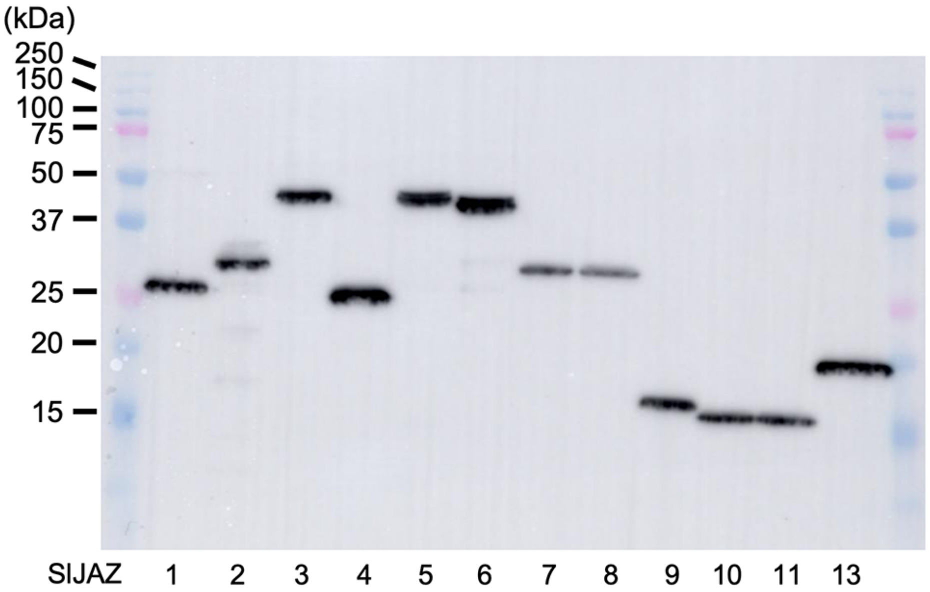

Figure S1. Expression of FLAG-*Sl*JAZ1-11/13 proteins by a wheat germ-derived cell-free protein expression system; *Sl*JAZ1: 24.9 kDa, *Sl*JAZ2: 29.2 kDa, *Sl*JAZ3: 34.7 kDa, *Sl*JAZ4: 23.4 kDa, *Sl*JAZ5: 41.1 kDa, *Sl*JAZ6: 33.4 kDa, *Sl*JAZ7: 27.4 kDa, *Sl*JAZ8: 28.1 kDa, *Sl*JAZ9: 13.8 kDa, *Sl*JAZ10: 13.1 kDa, *Sl*JAZ11: 15.6 kDa, *Sl*JAZ13: 16.1 kDa, respectively.

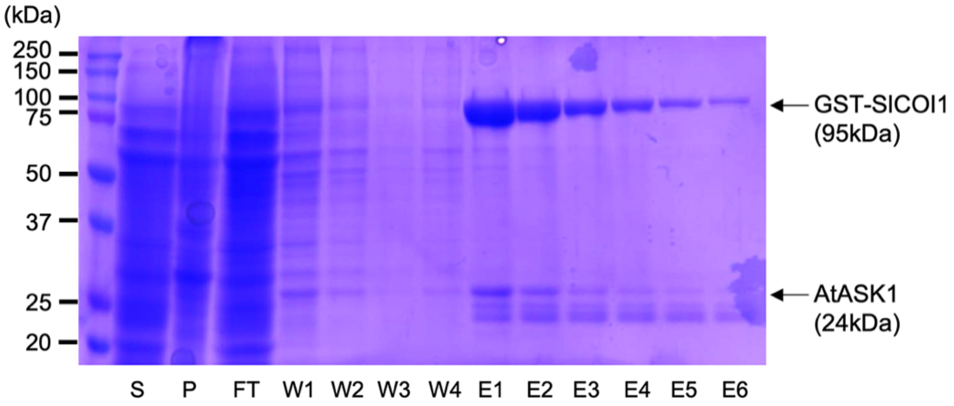

Figure S2. Expression and purification of GST-*Sl*COI1 protein by a cultured insect cell protein expression system (S: soluble fraction of cell lysate, P: insoluble fraction (pellet) of cell lysate, FT: flow-through fraction, W1-W4: washed fraction, E1-E6: elution fraction in glutathione-S-transferase affinity column chromatography, respectively).

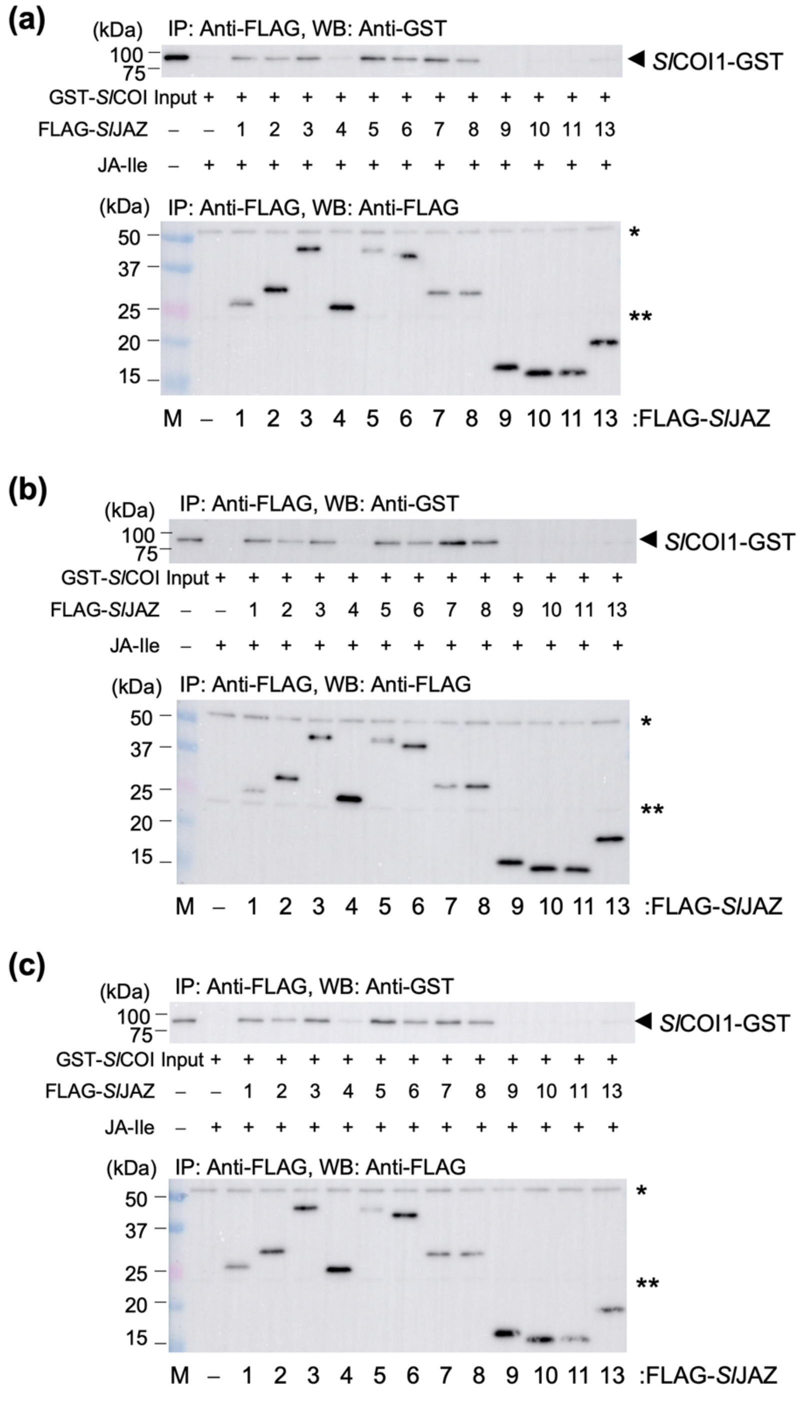

Figure S3. Results of three independent experiments (a-c for each result) shown in **Figure 2a**. Pull down assay of GST-*Sl*COI1 with FLAG-*Sl*JAZ (full-length proteins) in the presence of JA-Ile (100 nM) (top: anti-GST-HRP conjugate for detection of GST-*Sl*COI1, bottom: anti-FLAG antibody and anti-mouse-IgG HRP conjugate for detection of FLAG-*Sl*JAZs). * or ** show the bands derived from heavy chain or light chain of the anti-FLAG antibody.

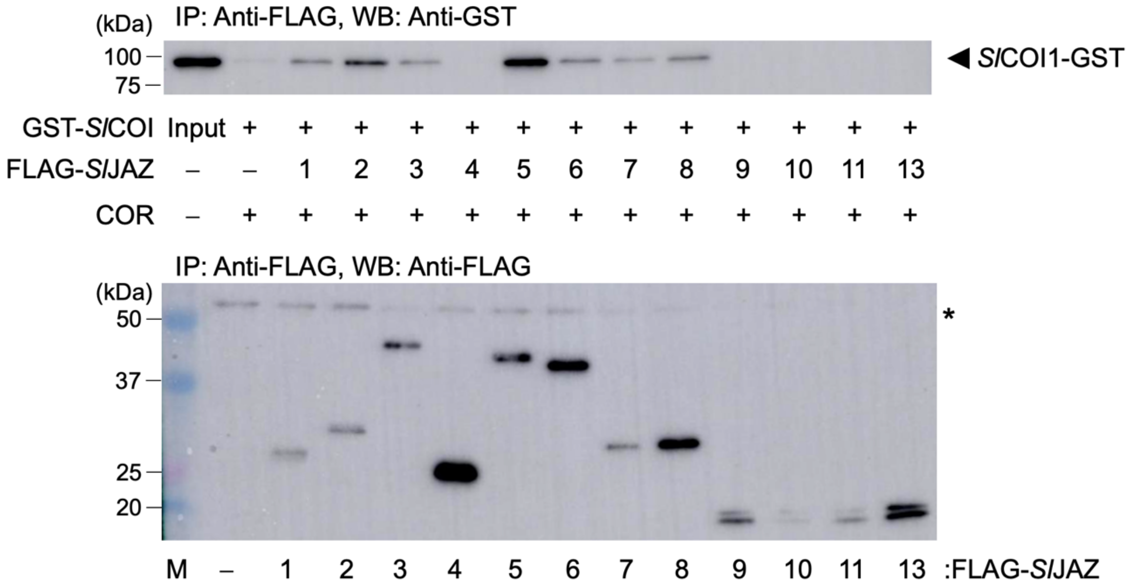

Figure S4. Pull down assay of GST-*Sl*COI1 with FLAG-*Sl*JAZ (full-length proteins) in the presence of COR (100 nM). GST-*Sl*COI1 bound to FLAG-*Sl*JAZ proteins was pulled down with anti-FLAG antibody and Protein G magnetic beads, and analyzed by immunoblotting (top: anti-GST-HRP conjugate for detection of GST-*Sl*COI1, bottom: anti-FLAG antibody and anti-mouse-IgG HRP conjugate for detection of FLAG-*Sl*JAZs). * shows the bands derived from heavy chain of the anti-FLAG antibody.

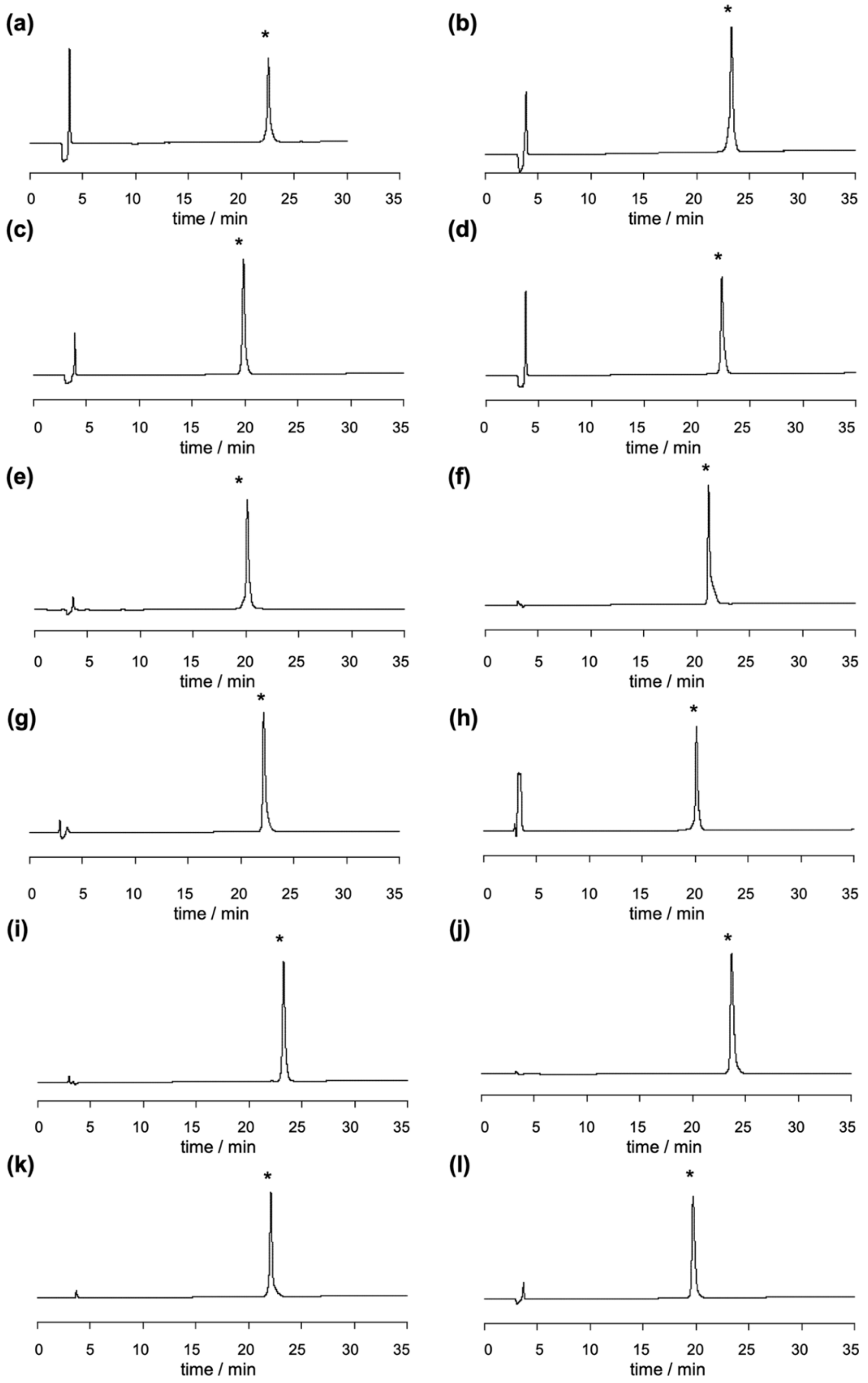

Figure S5. HPLC charts of purified fluorescein-conjugated *Sl*JAZ peptides; (a) Fl-*Sl*JAZ1, (b) Fl-*Sl*JAZ2, (c) Fl-*Sl*JAZ3, (d) Fl-*Sl*JAZ4, (e) Fl-*Sl*JAZ5, (f) Fl-*Sl*JAZ6, (g) Fl-*Sl*JAZ7, (h) Fl-*Sl*JAZ8, (i) Fl-*Sl*JAZ9, (j) Fl-*Sl*JAZ10, (k) Fl-*Sl*JAZ11, (l) Fl-*Sl*JAZ13. These figures were created by KaleidaGraph 4.1.1 (Synergy, Software, US).

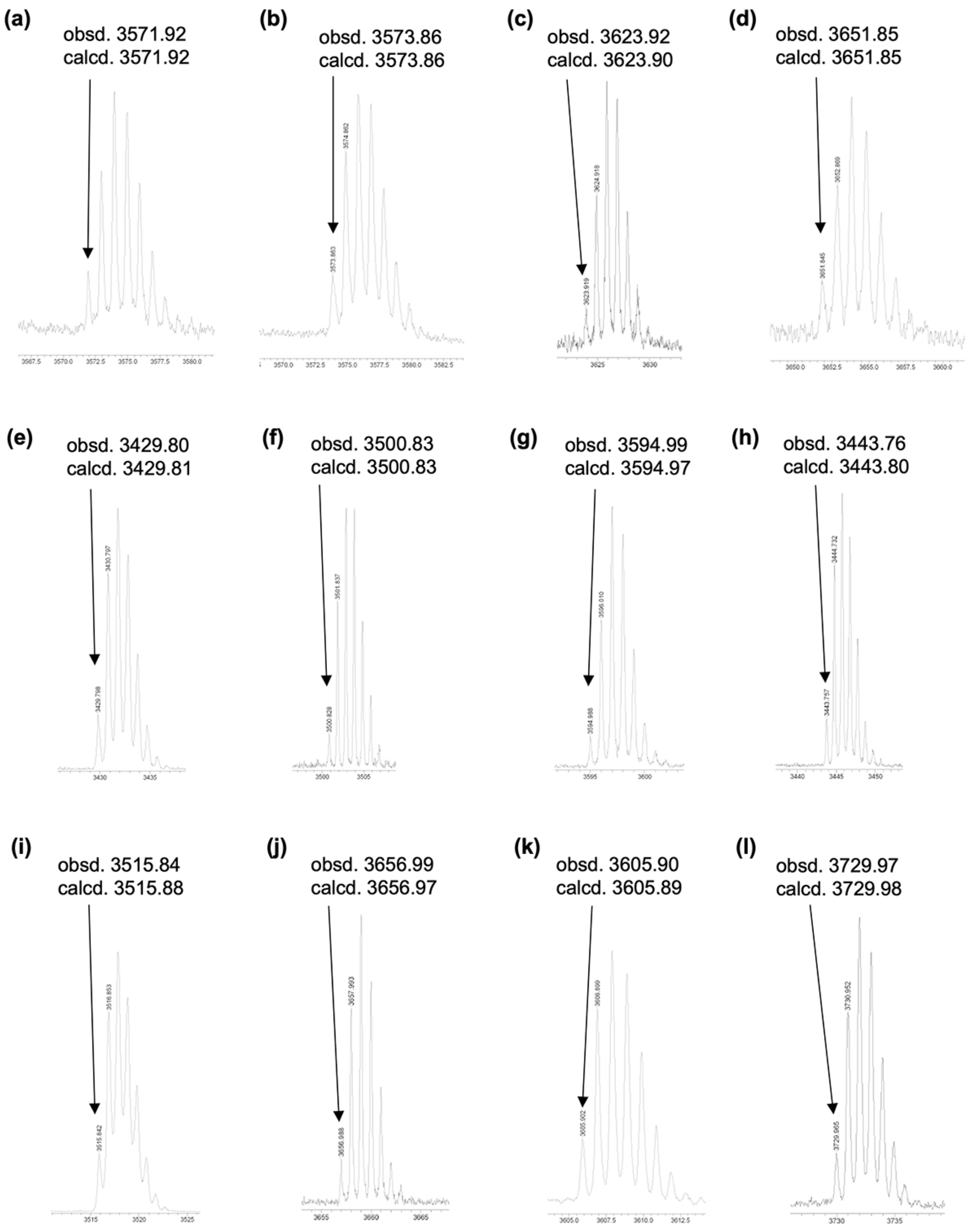

Figure S6. MALDI-TOF mass spectra of fluorescein-conjugated *Sl*JAZ peptides; (a) Fl-*Sl*JAZ1, (b) Fl-*Sl*JAZ2, (c) Fl-*Sl*JAZ3, (d) Fl-*Sl*JAZ4, (e) Fl-*Sl*JAZ5, (f) Fl-*Sl*JAZ6, (g) Fl-*Sl*JAZ7, (h) Fl-*Sl*JAZ8, (i) Fl-*Sl*JAZ9, (j) Fl-*Sl*JAZ10, (k) Fl-*Sl*JAZ11, (l) Fl-*Sl*JAZ13.

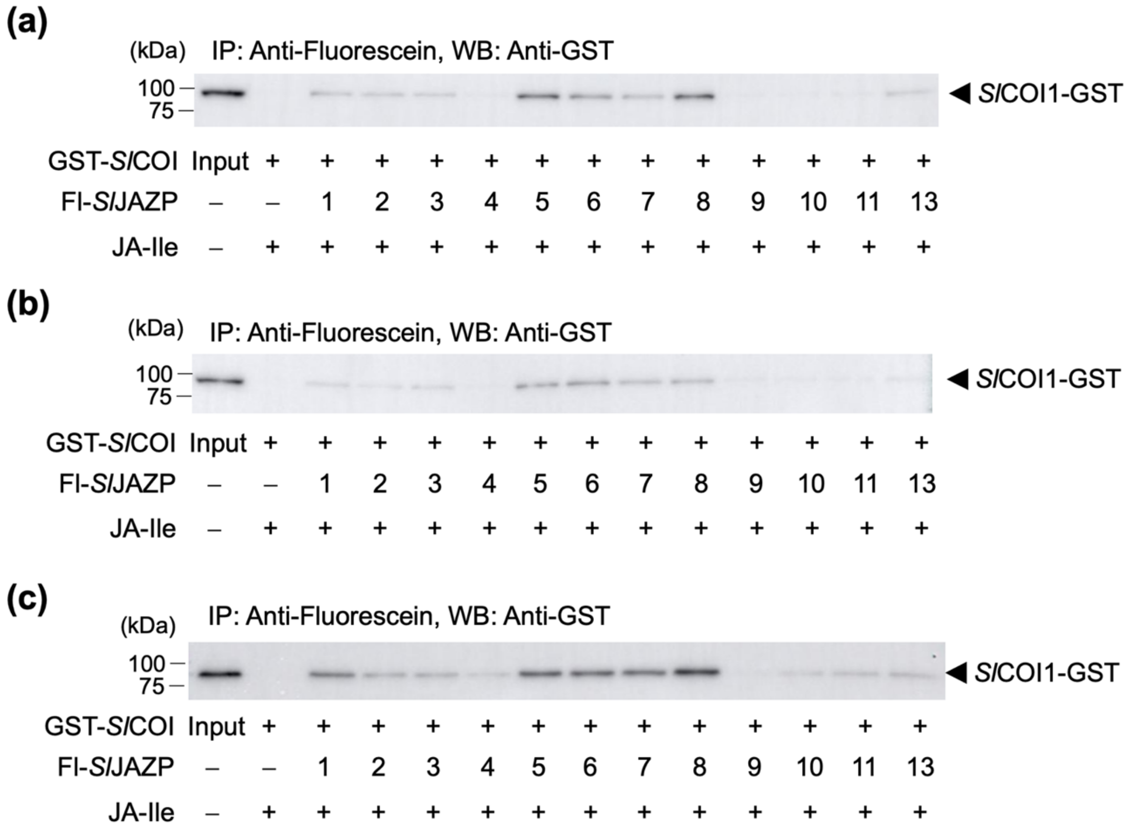

Figure S7. Results of three independent experiments shown in **Figure2b** (**a-c** for each result). Pull down assay of GST-*Sl*COI1 with Fl-*Sl*JAZPs in the presence of JA-Ile (100 nM).

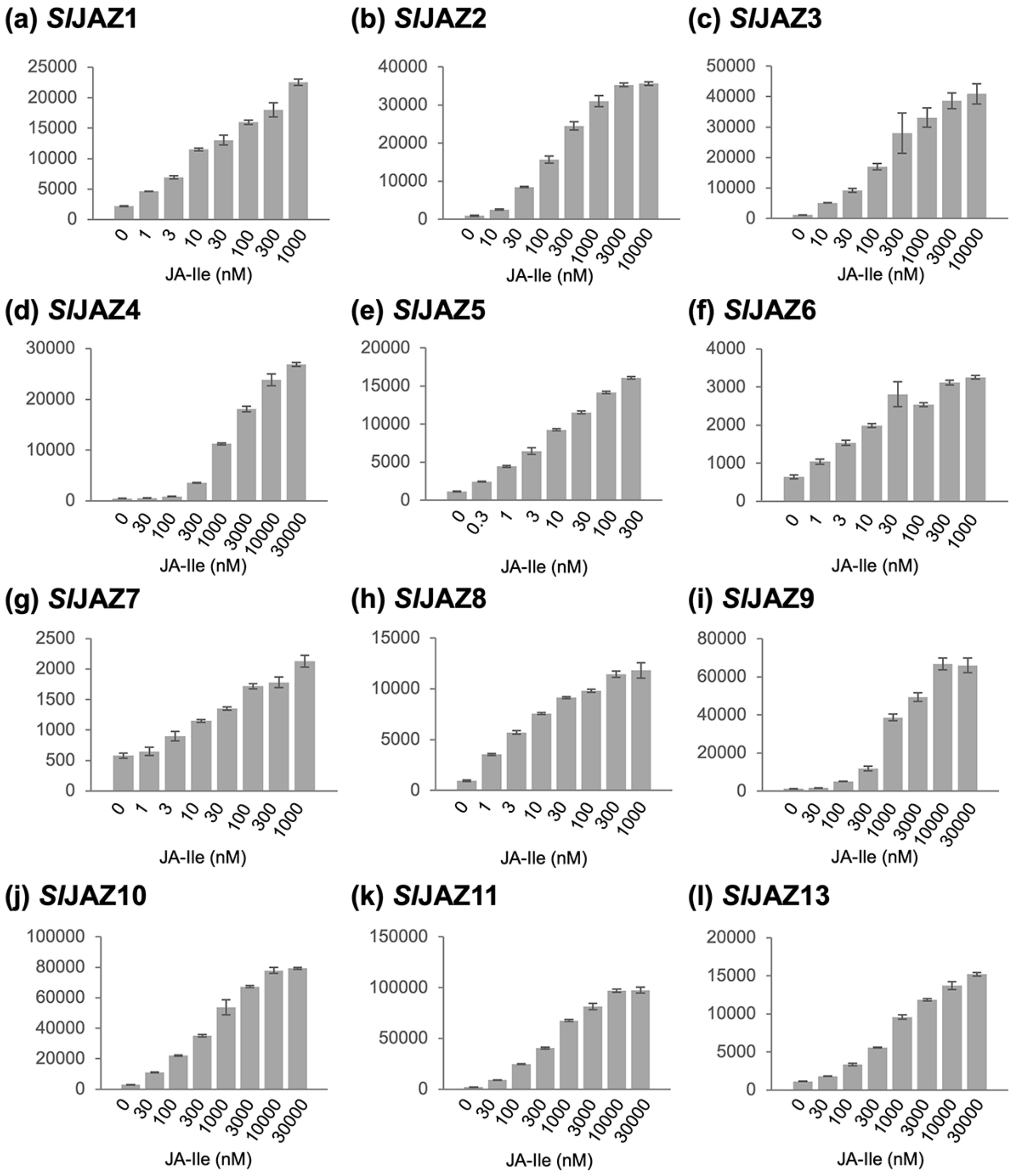

Figure S8. Signal intensity changes in the AlphaScreen assays using Fl-*Sl*JAZPs and GST-*Sl*COI1 with JA-Ile (0 – 30 μM). Experiments were performed in triplicate to obtain mean and S.D. (shown as error bars).

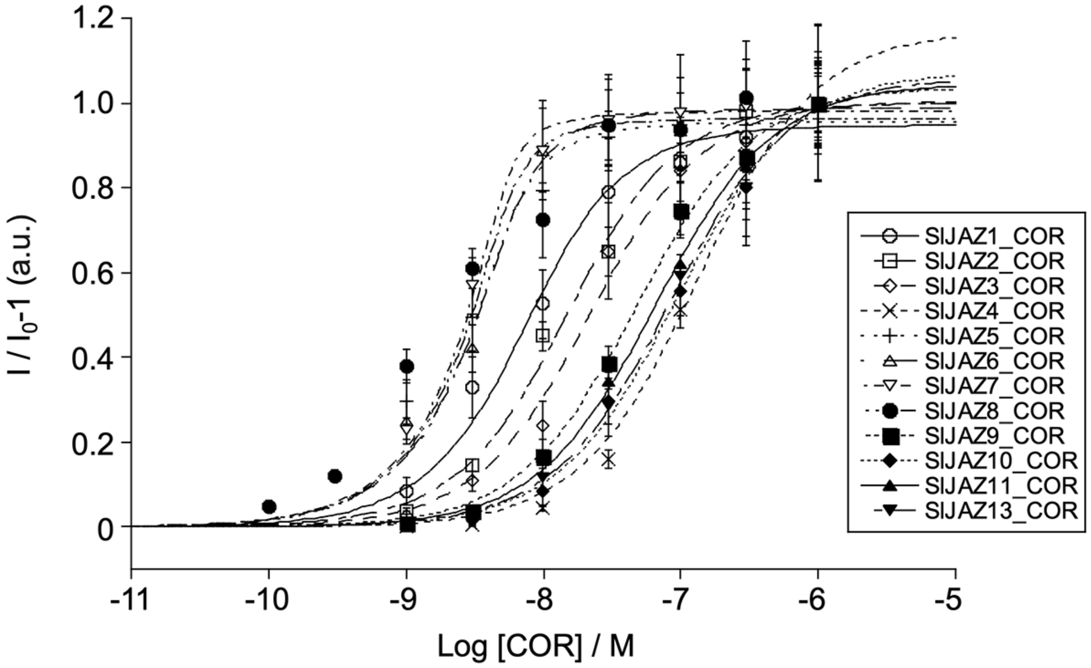

Figure S9. AlphaScreen assays using Fl-*Sl*JAZPs and GST-*Sl*COI1 with COR (0 – 1 μM). Experiments were performed in triplicate to obtain mean and S.D. (shown as error bars). This figure was created by KaleidaGraph 4.1.1 (Synergy, Software, US).

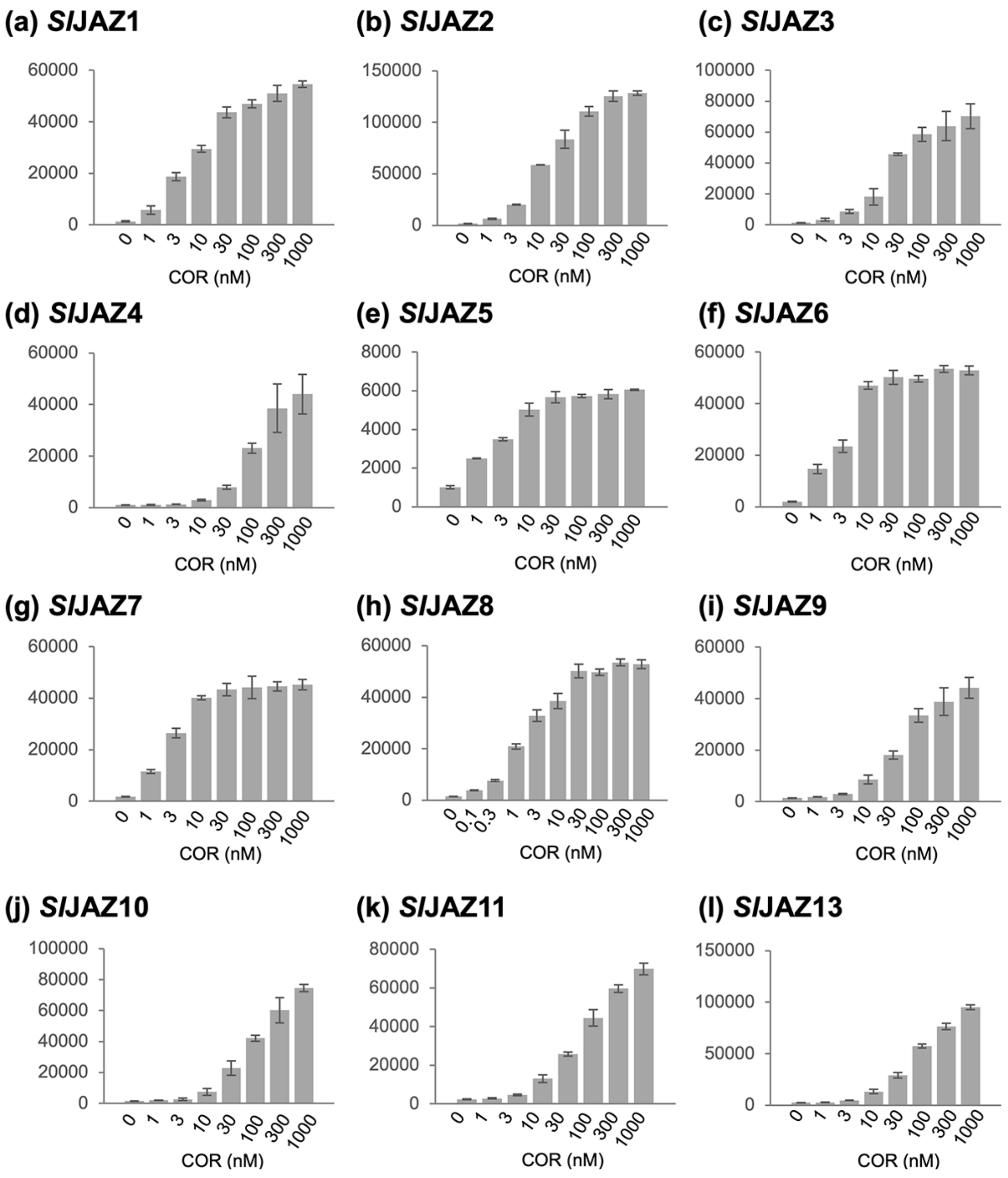

Figure S10. Signal intensity changes in the AlphaScreen assays using Fl-*Sl*JAZPs and GST-*Sl*COI1 with COR (0 – 1 μM). Experiments were performed in triplicate to obtain mean and S.D. (shown as error bars). These figures were created by KaleidaGraph 4.1.1 (Synergy, Software, US).

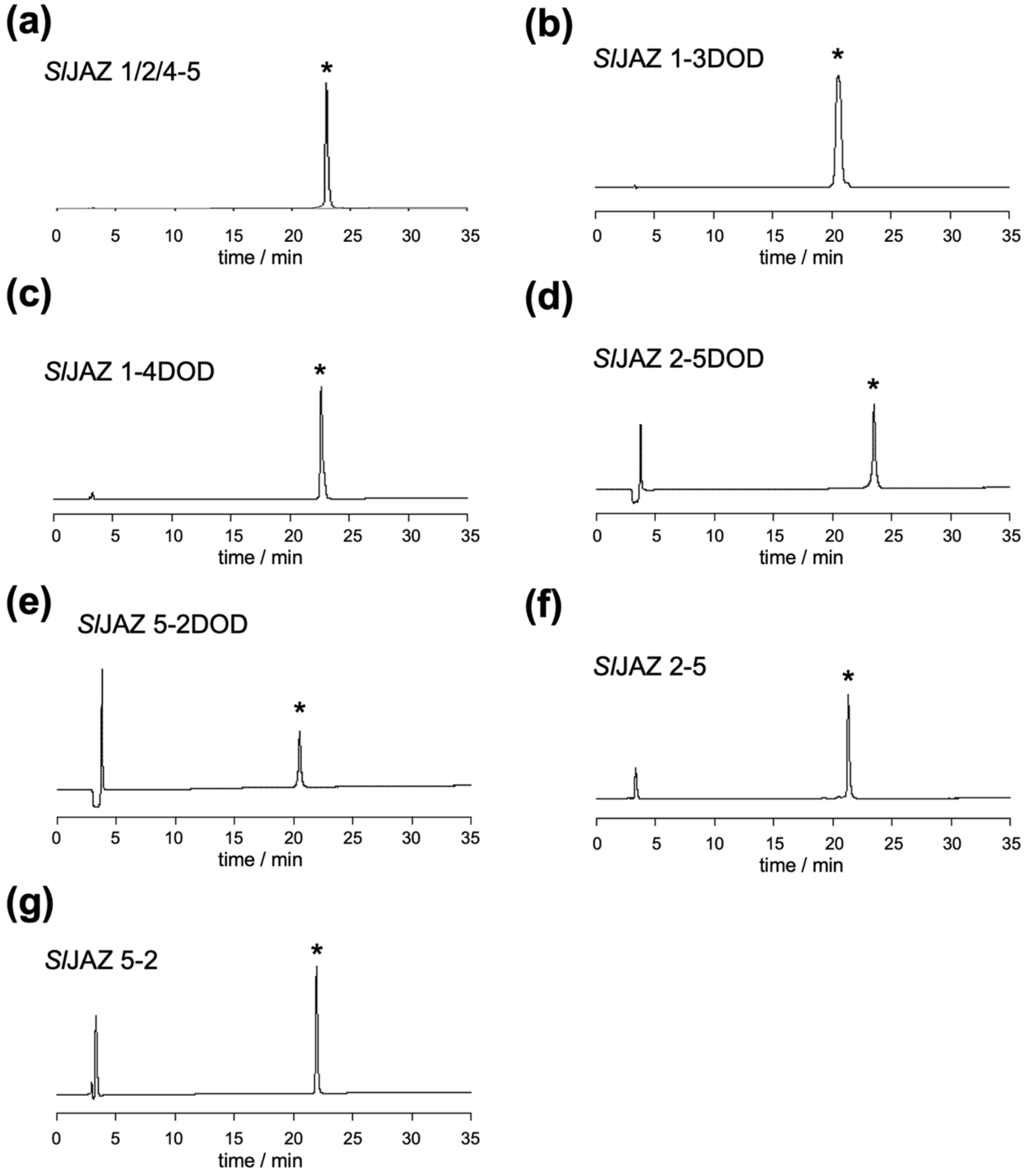

Figure S11. HPLC charts of the purified swapped Fl-*Sl*JAZPs. These figures were created by KaleidaGraph 4.1.1 (Synergy, Software, US).

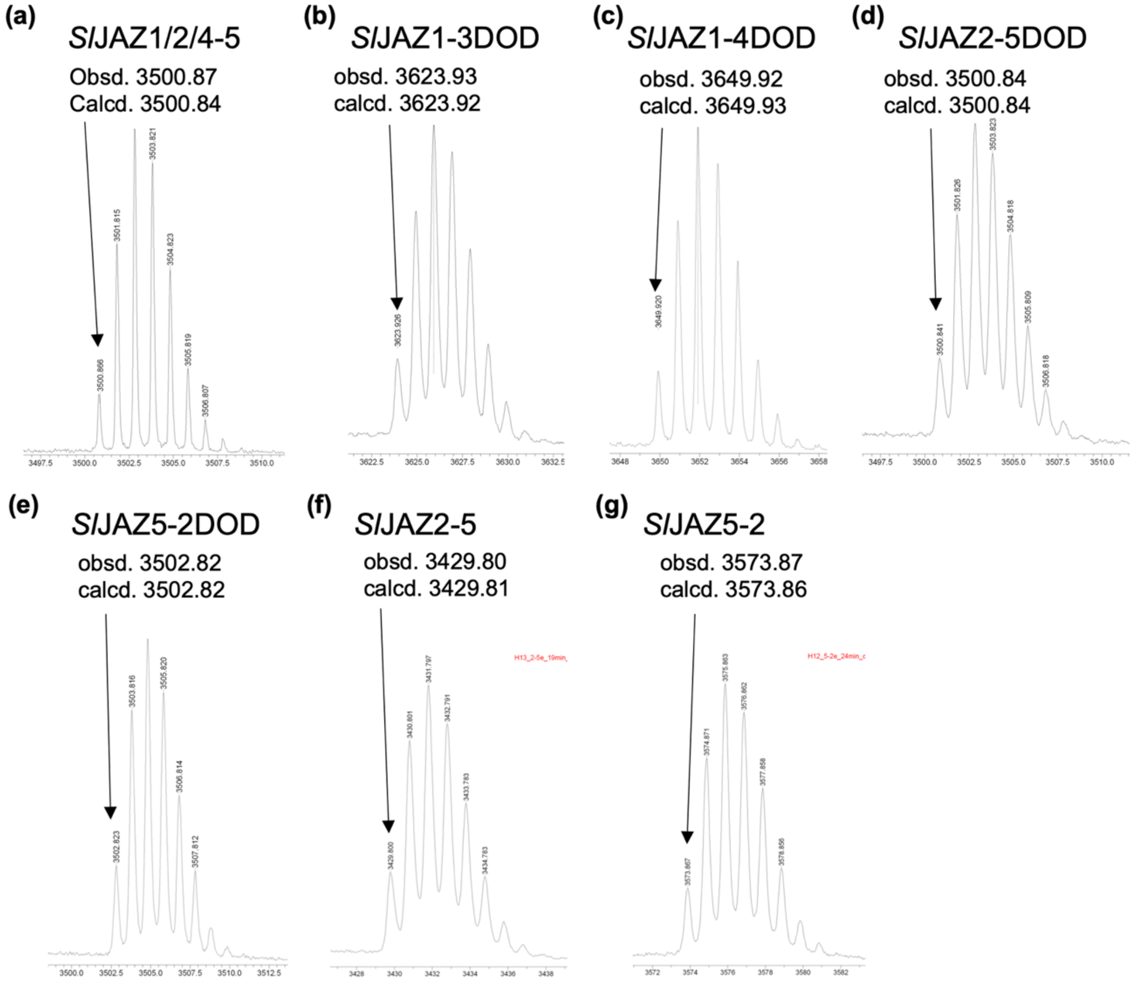

Figure S12. MALDI-TOF mass spectra of fluorescein-conjugated swapped *Sl*JAZ peptides.

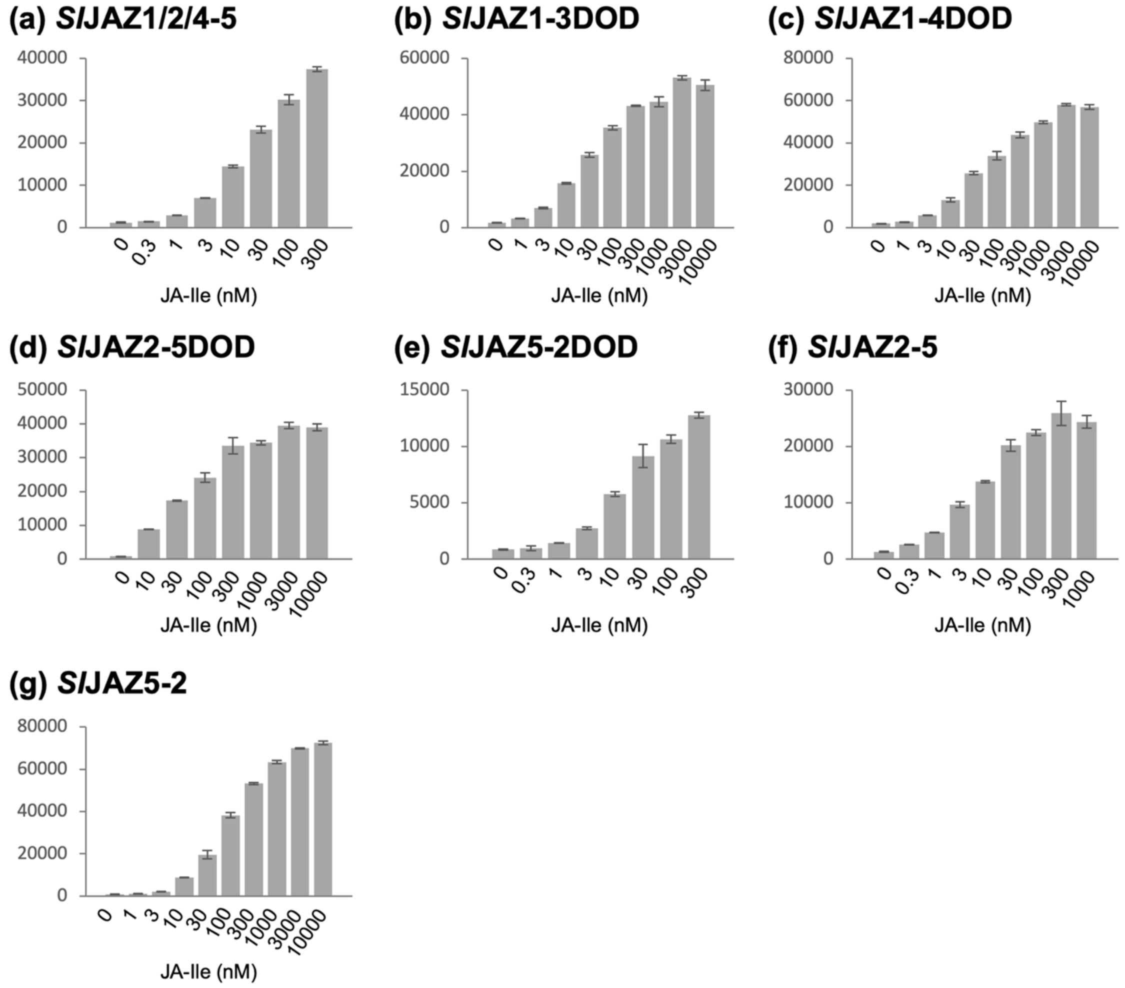

Figure S13. Signal intensity changes in the AlphaScreen assays using swapped Fl-*Sl*JAZPs and GST-*Sl*COI1 with JA-Ile (0 – 10 μM). Experiments were performed in triplicate to obtain mean and S.D. (shown as error bars).

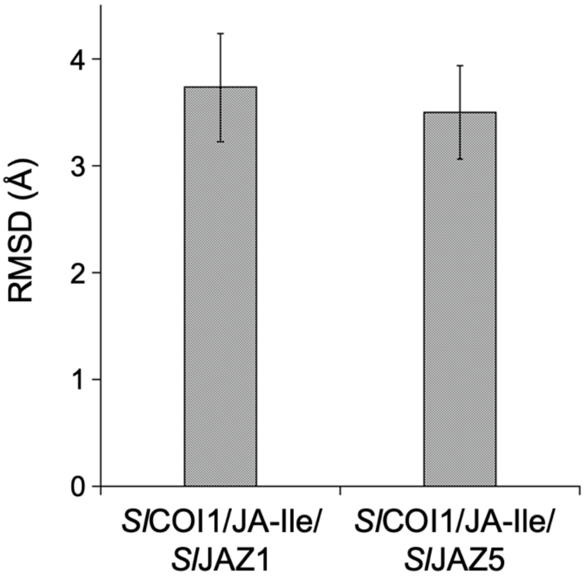

Figure S14. RMSD values of the final points (100 ns) of five independent MD simulations of *Sl*COI1/JA-Ile/*Sl*JAZ1 and *Sl*COI1/JA-Ile/*Sl*JAZ5 (shown in Figure 6a). Statistical analyses were done by Student's t-test (there was no significant difference between them (p = 0.461). These figures were created by KaleidaGraph 4.1.1 (Synergy, Software, US).

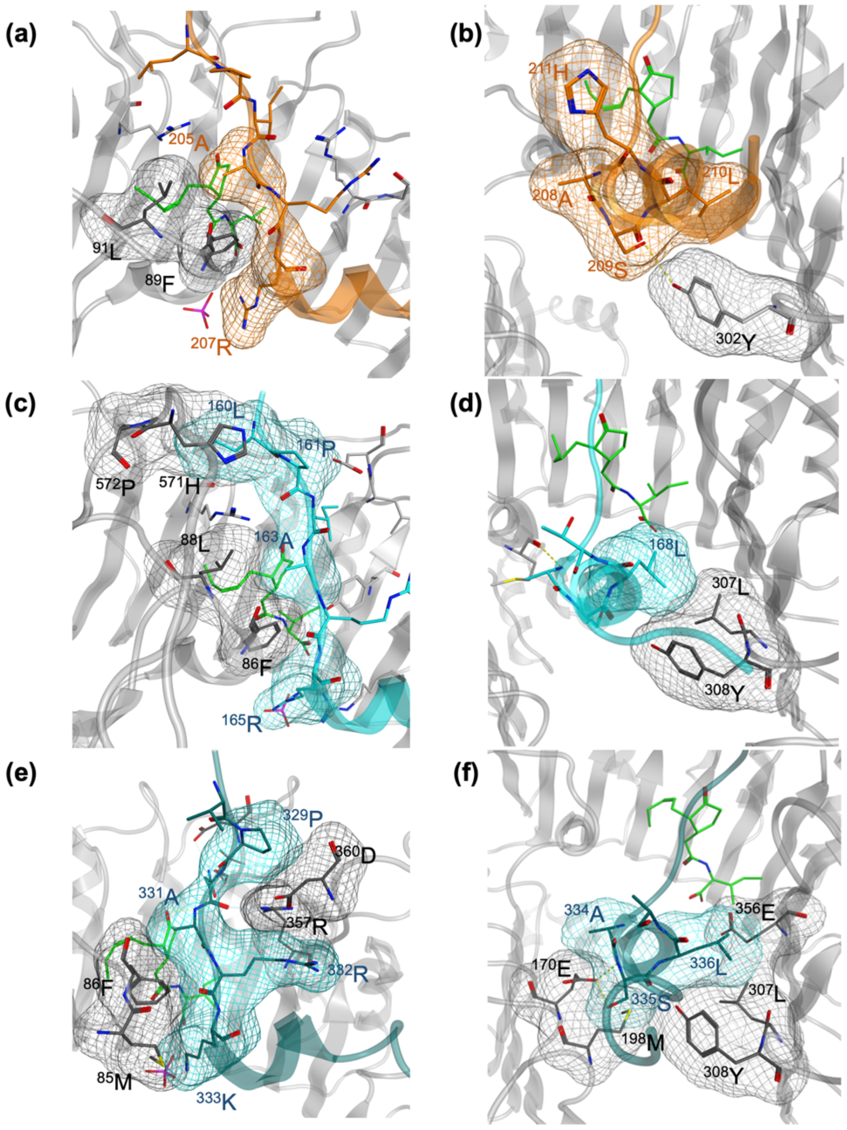

Figure S15. In silico analyses of *At*COI1-JA-Ile-*At*JAZ1 (PDB ID: 3OGL, **a, b**), *Sl*COI1-JA-Ile-*Sl*JAZ1 **(c, d)**, and *Sl*COI1-JA-Ile-*Sl*JAZ5 **(e, f)** to show the hydrophobic interaction between *Sl*COI1 and the extended degron of *Sl*JAZ. **(a)** The reported structure around the degron sequence of *At*JAZ1 in the complex *At*COI1-JA-Ile-*At*JAZ1. **(b)** The reported structure around the DOD sequence of *At*JAZ1 in the complex *At*COI1-JA-Ile-*At*JAZ1. **(c, e)** The obtained MD structure around the degron sequence of *Sl*JAZ in the complex *Sl*COI1-JA-Ile-*Sl*JAZ1 **(c)** or *Sl*COI1-JA-Ile-*Sl*JAZ5 **(e)**. **(d, f)** The obtained MD structure around the DOD sequence of *Sl*JAZ in the complex *Sl*COI1-JA-Ile-*Sl*JAZ1 **(d)** or *Sl*COI1-JA-Ile-*Sl*JAZ5 **(f)**. Electron densities of *At*/*Sl*COI1, *At*JAZ1, and *Sl*JAZ1/5 are represented with gray, orange, and cyan-colored mesh, respectively. These images were created by MOE 2020.09.

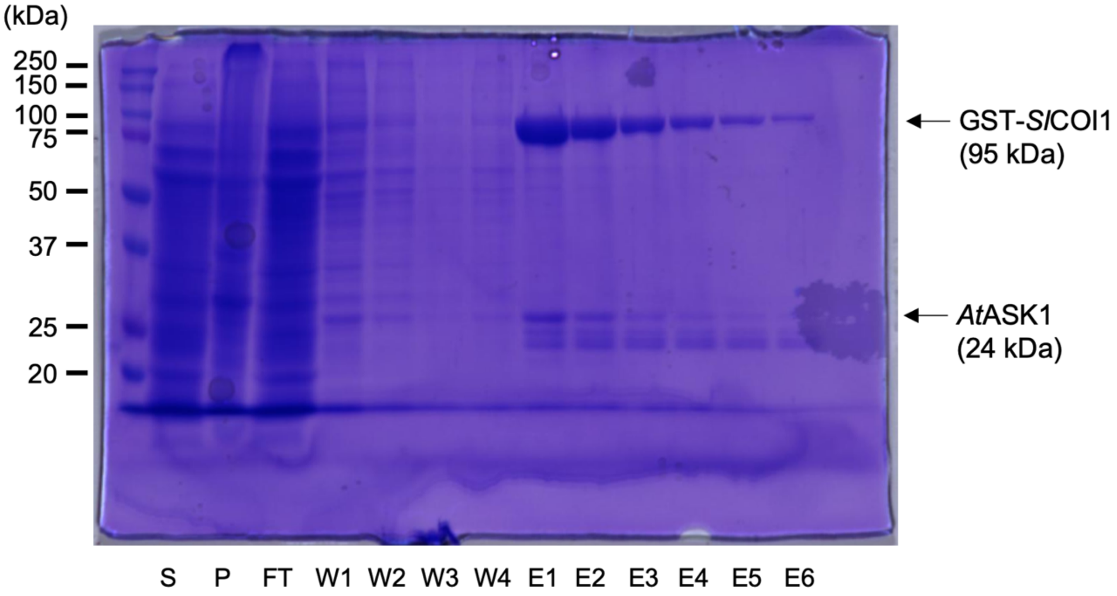

Figure S16. Uncropped image of **Figure S2**. Expression and purification of GST-*Sl*COI1 protein by cultured insect cell protein expression system (S: soluble fraction of cell lysate, P: insoluble fraction (pellet) of cell lysate, FT: flow-through fraction, W1-W4: washed fraction, E1-E6: elution fraction in glutathione-S-transferase affinity column chromatography, respectively).

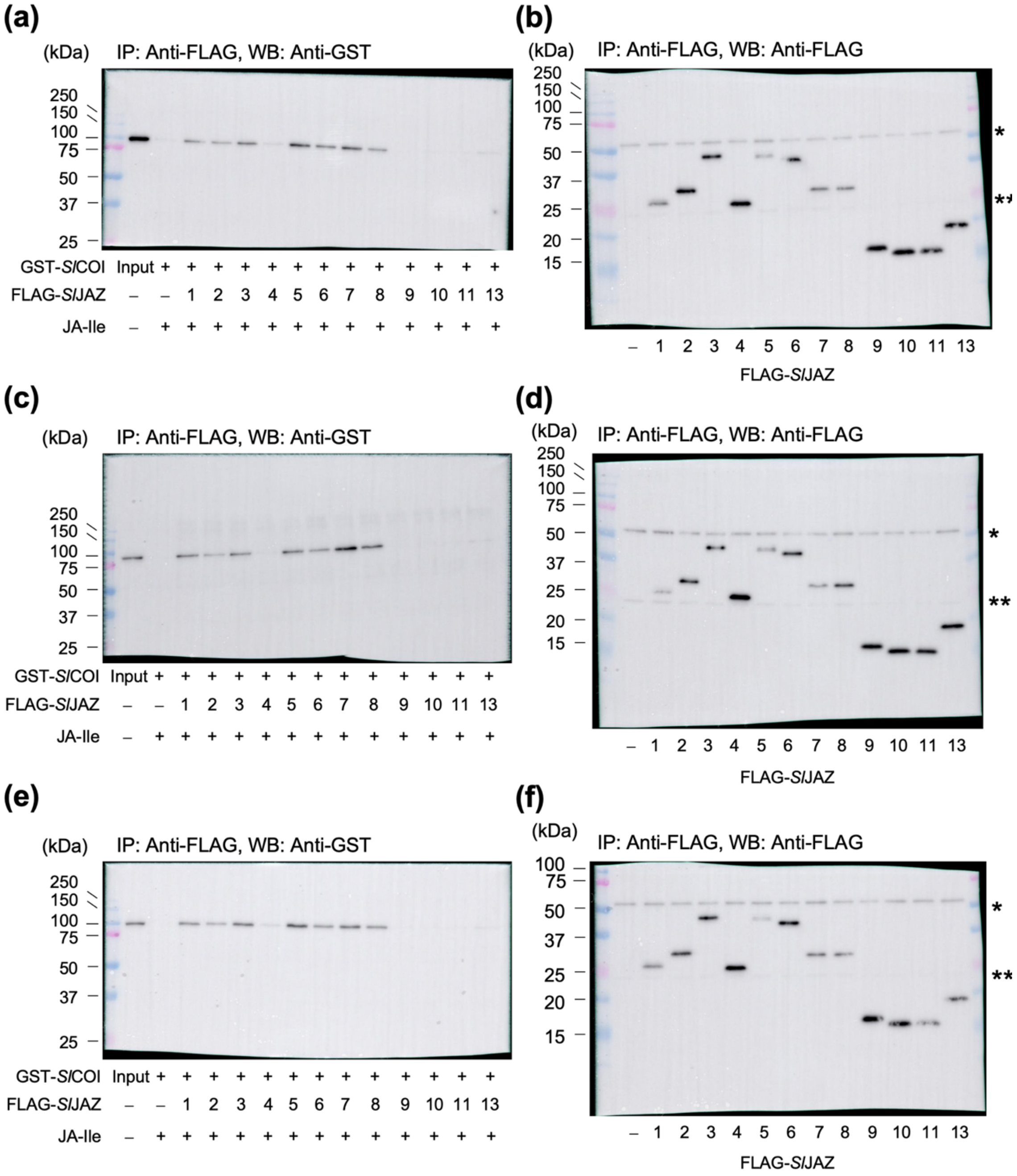

Figure S17. Uncropped images of **Figure S3** (**ab** for **Fig. S3a**, **cd** for **Fig. S3b**, and **ef** for **Fig. S3c**, respectively). Pull down assay of GST-*Sl*COI1 with FLAG-*Sl*JAZ (full-length proteins) and JA-Ile (100 nM) (**a**, **c**, **e:** anti-GST-HRP conjugate for detection of GST-*Sl*COI1, **b**, **d**, **f**: anti-FLAG antibody and anti-mouse-IgG HRP conjugate for detection of FLAG-*Sl*JAZs). * or ** show the bands derived from heavy chain or light chain of the anti-FLAG antibody.

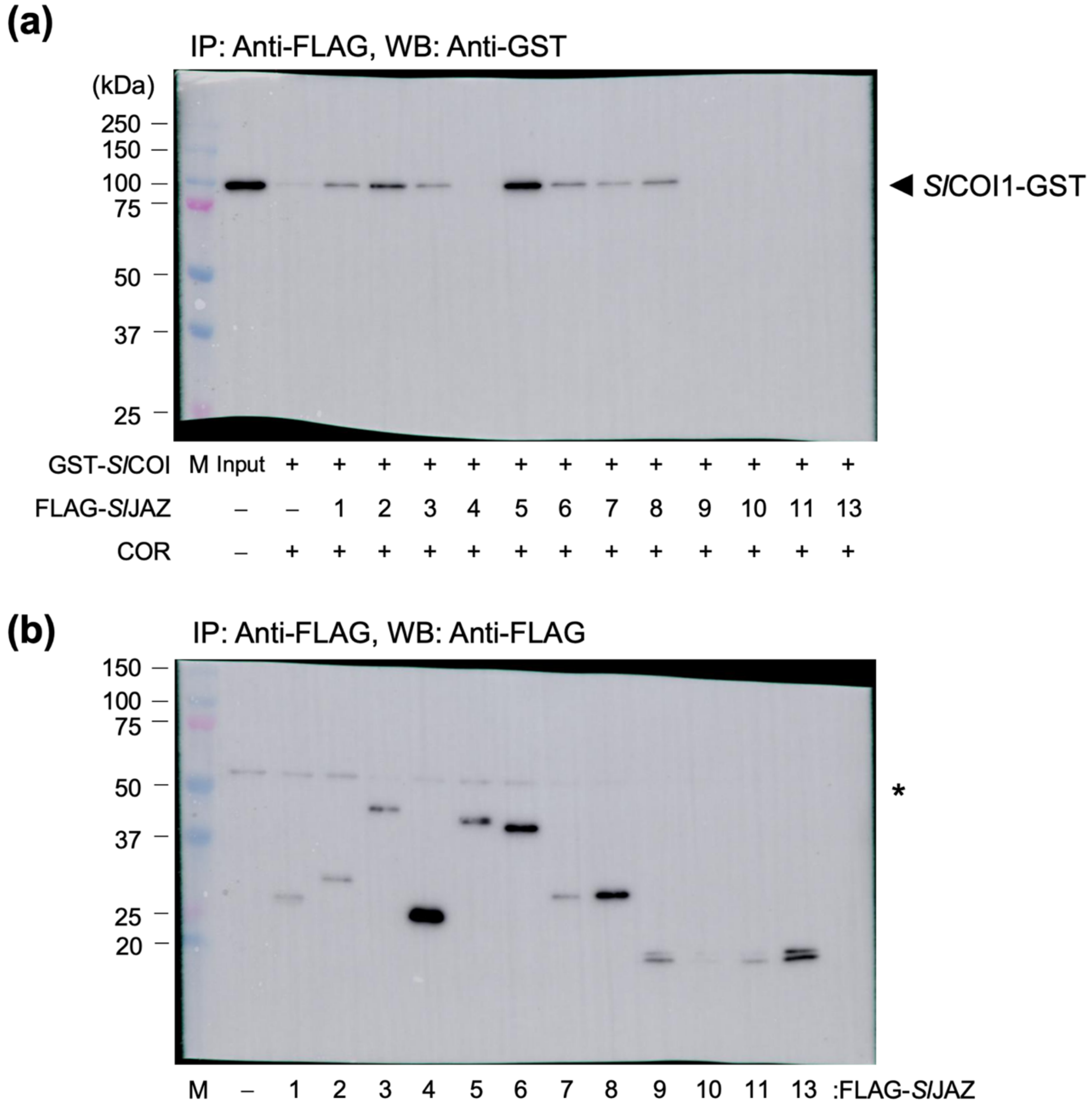

Figure S18. Uncropped blots of **Figure S4**. Pull down assay of GST-*Sl*COI1 with FLAG-*Sl*JAZ (full-length proteins) in the presence of COR (100 nM) (**a:** anti-GST-HRP conjugate for detection of GST-*Sl*COI1, **b**: anti-FLAG antibody and anti-mouse-IgG HRP conjugate for detection of FLAG-*Sl*JAZs). * shows the bands derived from heavy chain of the anti-FLAG antibody.

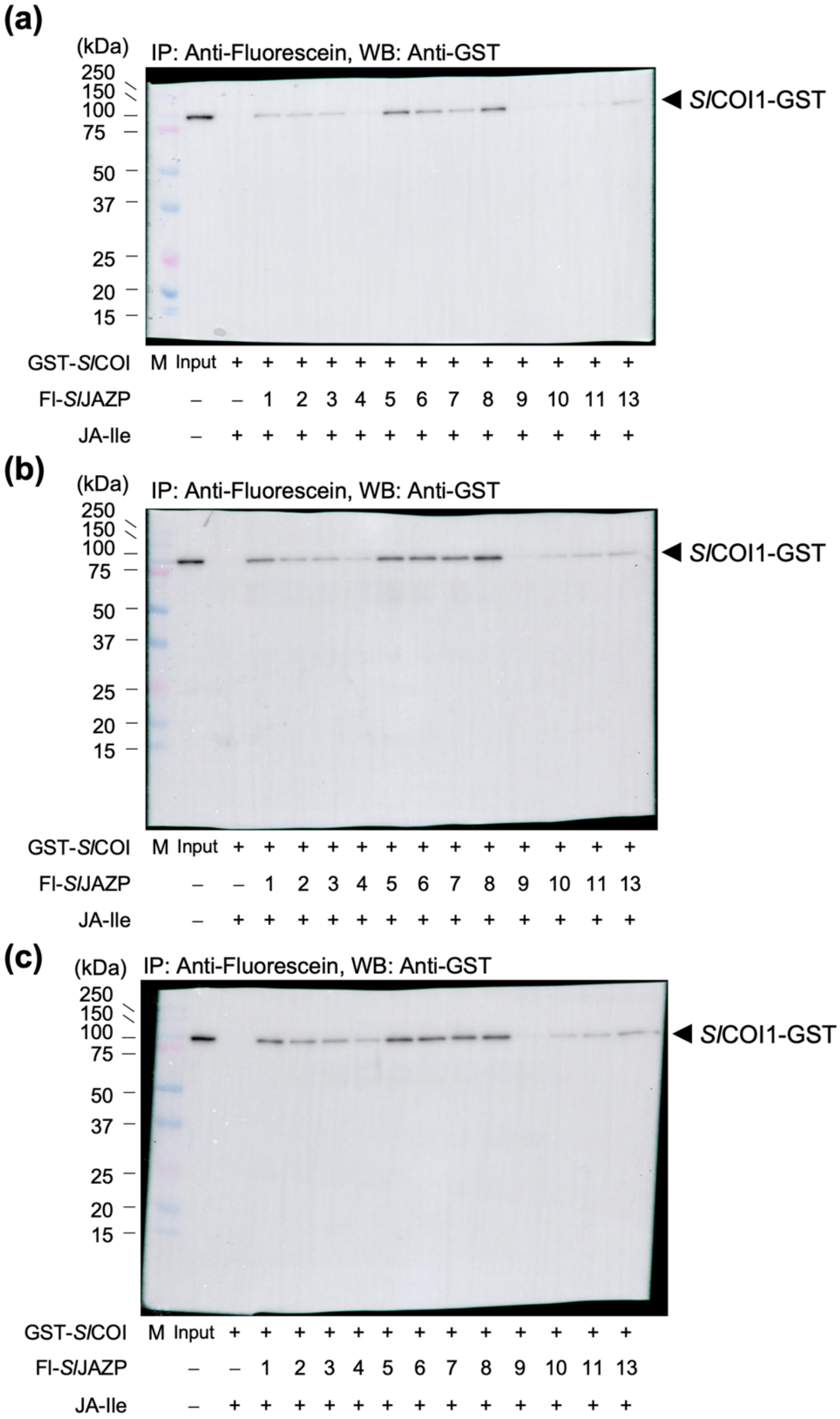

Figure S19. Uncropped blots of **Figure S4**. Pull down assay of GST-*Sl*COI1 with FLAG-*Sl*JAZ (full-length proteins) in the presence of COR (100 nM) (**a:** anti-GST-HRP conjugate for detection of GST-*Sl*COI1, **b**: anti-FLAG antibody and anti-mouse-IgG HRP conjugate for detection of FLAG-*Sl*JAZs). * shows the bands derived from heavy chain of the anti-FLAG antibody.

Table S1. Gene sequences of all primers for cloning used in this study.

*Sl*JAZ1	5′- CACCATGGCTTCATCGGAGATTGTGGATTCC -3′
	5′- CTAGTATTGCTCAGTTTTCACTGCAAATTGACCAC -3′
*Sl*JAZ2	5′- GGGGACAAGTTTGTACAAAAAAGCAGGCTTCATGGGGTCATCGGAAAATA -3’
	5′- GGGGACCACTTTGTACAAGAAAGCTGGGTCCTAGAAATATTGCTCAGTT -3′
*Sl*JAZ3	5′- GGGGACAAGTTTGTACAAAAAAGCAGGCTTCATGTCGAATTTATGTGACG -3′
	5′- GGGGACCACTTTGTACAAGAAAGCTGGGTCCTATAACTTGAAATTGAGA -3′
*Sl*JAZ4	5′- CACCATGTCAAATAGGCAACTTTGTTCATTAGATA -3′
	5′- CTAGAAATTGAGATCAAAATGATCTCCACG -3′
*Sl*JAZ5	5′- GGGGACAAGTTTGTACAAAAAAGCAGGCTTCATGGAGAGAGATTTCATGG -3′
	5′-

	GGGGACCACTTTGTACAAGAAAGCTGGGTCCTACTTGACCAAACTGATT -3′
*Sl*JAZ6	5′-GGGGACAAGTTTGTACAAAAAAGCAGGCTTCATGGAGAGGGACTTTATGG -3′
	5′-GGGGACCACTTTGTACAAGAAAGCTGGGTCCTAGGTCTCCTTACCGGCT -3′
*Sl*JAZ7	5′-GGGGACAAGTTTGTACAAAAAAGCAGGCTTCATGGATTCAAGAATGGAGA -3′
	5′-GGGGACCACTTTGTACAAGAAAGCTGGGTCTTAGTTTTCCCAATGAACG -3′
